# Recent Advances in Recoverable Systems for the Copper-Catalyzed Azide-Alkyne Cycloaddition Reaction (CuAAC)

**DOI:** 10.3390/molecules21091174

**Published:** 2016-09-05

**Authors:** Alessandro Mandoli

**Affiliations:** 1 Dipartimento di Chimica e Chimica Industriale Università di Pisa, Via G. Moruzzi 13, Pisa 56124, Italy; alessandro.mandoli@unipi.it; Tel.: +39-050-221-9280; 2 ISTM-CNR, Via C. Golgi 19, Milano 20133, Italy

**Keywords:** supported catalysts, click-chemistry, continuous-flow chemistry, 1,2,3-triazoles

## Abstract

The explosively-growing applications of the Cu-catalyzed Huisgen 1,3-dipolar cycloaddition reaction between organic azides and alkynes (CuAAC) have stimulated an impressive number of reports, in the last years, focusing on recoverable variants of the homogeneous or quasi-homogeneous catalysts. Recent advances in the field are reviewed, with particular emphasis on systems immobilized onto polymeric organic or inorganic supports.

## 1. Introduction

Efforts towards selectivity, atom economy, generation of molecular diversity, and experimental convenience have been a constant drive in modern organic synthesis. Still, their union in the context of bond-forming (”ligation”) processes, with the conceptualization of ”click-chemistry“ by Kolb, Finn, and Sharpless [1], appears to have become a potent trigger of chemists’ inventiveness in recent years.

**Scheme 1:**
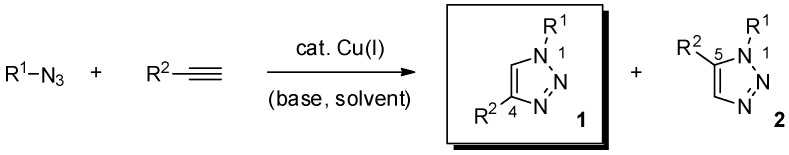
The copper-catalyzed azide-alkyne cycloaddition reaction (CuAAC).

Essential to this outcome was the parallel and independent introduction, by the groups of Meldal and Sharpless, of the copper-catalyzed azide-alkyne cycloaddition reaction (Scheme 1) [2,3]. In fact, the orthogonal character vs. the reactivity of most functional groups, complete regioselectivity in favor of the 1,4-disustituted-1,2,3-triazole product **1** (“triazole” hereafter, if not noted otherwise), mild reaction conditions, and easy installation of the required azide and terminal alkyne moieties in the reactive partners, render the metal-promoted version of the long-standing Huisgen 1,3-dipolar cycloaddition reaction the most prominent technique in the click-chemistry toolbox [4,5].

**Figure 1:**
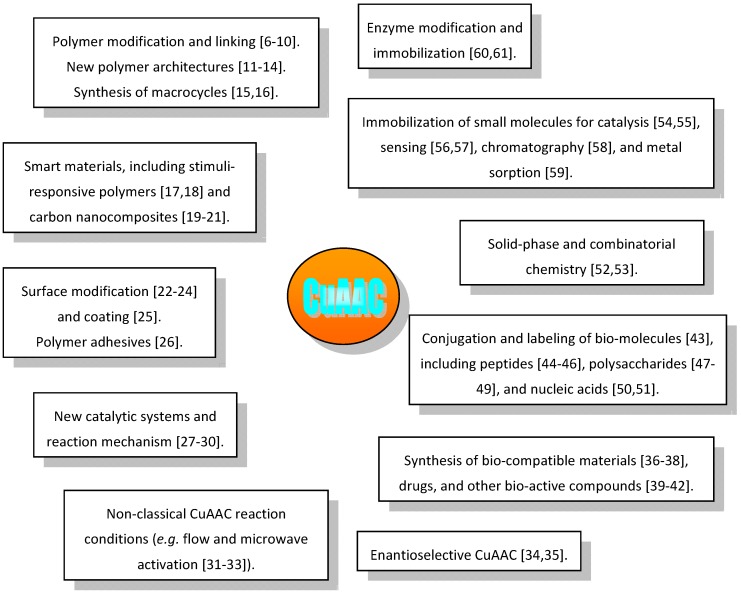
Main publication areas of CuAAC-related papers and selected examples of recent trends.

With applications ranging from material science to medicinal chemistry it is not surprising that investigations connected with the CuAAC topic, or just making use of this technique, are appearing with impressive and increasingly high frequency in the literature. As shown in Figure 1, these advancements comprise long-standing topics and some incompletely settled aspects like, e.g., the reaction mechanism, as well as new trends and examples of use of CuAAC in unprecedented directions [6,7,8,9,10,11,12,13,14,15,16,17,18,19,20,21,22,23,24,25,26,27,28,29,30,31,32,33,34,35,36,37,38,39,40,41,42,43,44,45,46,47,48,49,50,51,52,53,54,55,56,57,58,59,60,61].

**Figure 2:**
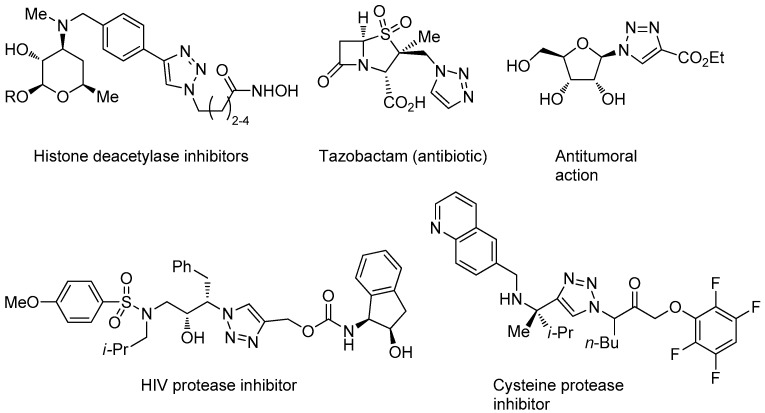
Selected examples of drugs and other bioactive compounds containing the 1,2,3-triazole nucleus.

In addition of being an effective way for linking together different molecules, the appreciation that the triazole structural motif may have pharmacologic relevance, e.g., as a more stable replacement for peptide bonds and in virtue of its dipole moment, hydrogen-bonding, and π-π stacking capabilities [42], improved further the interest towards CuAAC in the medicinal chemistry field. Even if less represented than the isomeric 1,2,4-triazole motif, contained in various approved drugs [62,63], examples of bio-active compounds that embed the 1,2,3-triazole nucleus keep appearing continuously in the literature or have already an established clinical use (Figure 2) [40,42].

Despite the many successes of CuAAC, the underlying chemistry suffers from three main drawbacks that pose limits to its usefulness, not only in a medicinal chemistry perspective but often also in the case of materials for special applications: (i) use of organic azides, which are not easy to purify and handle at scale because of their potential instability; (ii) contamination of the click product with catalyst components, copper above all; and (iii) instability of the Cu(I) oxidation state, which imposes inert-atmosphere handling techniques or the addition of an excess of a reducing agent like sodium ascorbate [64]. In the specific context of the modification of bio-molecules like, e.g., polysaccharides and polypeptides, further problems may arise then from the generation of highly reactive species (H_2_O_2_, hydroxyl radicals, and dehydroascorbic acid), which cause chain cleavage or denaturation [65,66,67].

**Scheme 2:**
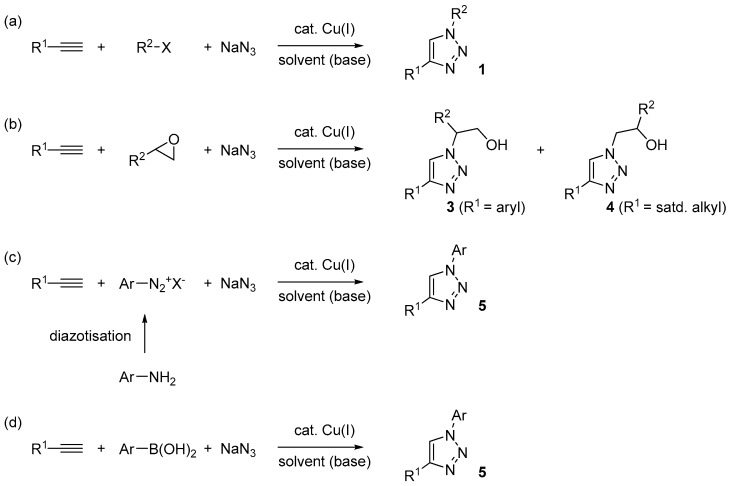
Three-components CuAAC reactions (**a**–**d**) discussed in this paper.

In addition to the use of flow-chemistry [32], a potentially general solution for the former problem is represented by in situ generation of the organic azide under multi-component reaction conditions [68]. In principle, with this approach it is possible to realize the consumption of the organic azide in the CuAAC step, as soon as it forms, and then avoid any build-up in the reaction mixture. Depending on the nature of the azide intermediate (e.g., aliphatic or aromatic) and of the starting material selected for the purpose, different possibilities exists in this respect. As summarized in Scheme 2, those met more frequently, and discussed in detail in the following sections, involve the use of:

- Alkyl halides (Scheme 2a), often of the “activated” type (benzyl, allyl, α-halocarbonyls) with respect to the nucleophilic substitution reaction;
- Epoxides (Scheme 2b), which can lead to the formation of differently substituted triazoles (**3** and **4**) in dependence of the regioselectivity of attack of azide ions on the oxirane ring;
- Aromatic diazonium salts (Scheme 2c), either commercially available (e.g., tetrafluoroborate salts) or produced in situ by diazotisation of the corresponding aromatic amine;
- Aromatic boronic acids (Scheme 2d), which may undergo azido-deboronation under the action of the same copper catalyst involved in the CuAAC process [69].

On the contrary, the problems associated with the use of the copper catalyst have been tackled by adopting widely different strategies, including the use of strained alkynes or highly reactive azides, that do not require metal catalysis at all [65,70,71,72], and reactants with chelating capability, which allow to reduce the catalyst loading [73]. Besides, many efforts were committed to the development of more effective catalytic systems based on copper or other metals [30]. Especially in the case of rhodium-catalyzed process, the latter choice may display a useful complementary profile with respect to CuAAC in that –with the proper choice of the catalytic complex-1,5-disubstituted-1,2,3-triazole products or even trisubstituted ones can be obtained from the reaction of terminal and internal alkynes, respectively. However, these alternatives still suffer from contamination of the product with transition metals and, usually, require more expensive catalyst precursor than CuAAC itself.

As far as copper is concerned, some of the shortcomings associated with traditional systems (e.g., CuX salts in the presence of an amine co-catalyst or CuSO_4_ with an excess of sodium ascorbate) were eliminated with the introduction of stabilizing ligands for the +I oxidation state of the metal and proper scavenging of reactive by-products [66,74]. Under these conditions CuAAC proceeds often under air, with catalyst concentration in the μM range and copper loading that, in some cases, can be reduced down to 0.01 mol % with respect to the substrates. Even so, the use of low-molecular weight soluble catalytic systems leaves unanswered the problem of contamination of the crude product with metal and, possibly, ligand residues. For instance, in the case above a copper content up to 25 ppm might be found in the crude from a CuAAC reaction forming a compound of average molecular weight. This concentration exceeds the metal amount often quoted as acceptable for pharmaceutical applications (15 ppm) [75].

Considering also that the use of 0.01 mol % of copper catalyst represents more the exception than the rule, especially when structurally complex triazoles have to be prepared, one may expect that such a threshold is surpassed by several order of magnitude when this chemistry is employed in scenarios of practical synthetic relevance. In this regard it is worth noting that while the possibility of running CuAAC reactions with catalysts loadings in the low ppm range has been reported with systems comprising self-assembled gel [76], dendrimeric ligands [77], and homogeneous catalysts comprising a molecularly-enlarged water-soluble tris-triazole ligand [78], demonstration of the scope of these approaches to molecules more complex of those usually employed for benchmarking new CuAAC protocols are just beginning to appear [78].

Together with the fact that copper removal can be difficult, in particular for highly functionalized or sensitive products [79], these problems tend to collide with the goal of prompt product purification underlying in the concept of click-chemistry. Therefore it is not surprising that many attempts have been described in the course of the years, aimed to separate the CuAAC catalytic system from the reaction mixture in more effective ways.

Some of these approaches rely on known homogeneous catalysts employed under alternative conditions like, e.g., recent reports making use of supercritical CO_2_ [80], glycerol, poly(ethylene glycol) (PEG), room-temperature ionic liquids, and their combinations thereof, as neoteric reaction media [81,82,83,84,85]. Similarly, water-soluble catalytic systems can provide an excellent opportunity for separation and recycling [78,86].

Albeit straightforward, this choice shows occasional limitations in terms of catalytic activity, seamless general implementation and, above all, effectiveness in reducing the residual copper content to an extent that might cope with the said regulatory requirements. In an alternative strategy, catalytic systems specifically designed for easier separation were therefore developed, typically by immobilization of copper species onto insoluble supports or quasi-homogeneous materials, like nanoparticles. Even if the degree of success may greatly vary from case to case, as discussed in the next sections, on the average this route appears better fit for the effective removal of the catalytic system from the reaction mixture and for the abatement of copper content in the crude by techniques amenable of use even at scale, like filtration, centrifugation, and magnetic decantation.

Of course, with this choice an opportunity arises also for catalyst recovery and reuse. In this respect it might be argued that, given the cheapness of most Cu(I/II) catalyst precursors, this goal is not necessarily the primary objective in the development of improved catalytic systems for CuAAC. Nevertheless, the possibility of recycling the metal species and any associated ligand, recovered “for free” at the product purification stage, is an appealing feature that stimulated much work in this direction.

The topic of recoverable systems for CuAAC has been briefly summarized before [87], up to the comprehensive reviews by Haldon et al. in 2015 [88], and Chassaing et al. [89], early this year. In addition, some specific aspects were covered in context like the use of supported ionic liquid phase catalyst [90], nanostructured and nanoporous Cu catalysts [91], CuO nanoparticles [92], and in the recent accounts by the groups of Alonso and of Astruc on the use of copper nanoparticles (CuNPs) [93], magnetic or dendritic catalysts [55], and present trends in azide-alkyne cycloaddition reactions [30].

Even so, the pace of research in the field of recoverable catalytic systems for CuAAC is so fast at present, that many more examples have appeared since the publication of the reference works above. The discussion of these recent advances, mostly published in the period 2015–mid 2016, is the aim of the present review.

In this regard, it must be pointed out that while recoverable systems for CuAAC will be covered in the broadest sense, the focus will be essentially on catalysts on insoluble supports and quasi-homogeneous systems where the presence of an organic polymer plays a decisive role. In general, an effort will be made for summarizing preparation method, characterization, conditions of use, activity, and scope of the various systems. Whenever possible, and for the reasons discussed above, the specific issues of catalyst leaching and product contamination will be examined in detail.

## 2. Cu, Cu_2_O, CuO, and CuX Nanoparticles and Nanocomposites

Like in many other catalytic processes [94], nanoparticles have received a great deal of attention in the context of CuAAC. Although just a fraction of the metal centers are actually exposed to the surrounding reaction medium (e.g., 28% for a spherical CuO nanoparticle with a diameter *d*_p_ = 4.7 nm) [95], nano-sized systems may nonetheless display high specific activity that, in some cases, appears related with facet index [96]. Because Cu_2_O is chemically unstable in air and Cu(0) nanoparticles tend to sinter or dissolve under CuAAC reaction conditions [93,97], some kind of stabilization is normally required. Similarly to the oxide layer covering bulk copper [75], the same problem is apparently met with porous forms of the metal and CuNPs prepared in solution, for which recent reports do not describe any recycle at all or show relatively fast loss of catalytic activity [98,99,100,101]. In many instances a solution to these problems is provided by the use of an insoluble support with suitable complexing sites, like amino groups [102], which may be very effective in preventing particle aggregation and extensive metal leaching.

### 2.1. Carbon Supports

Copper-in-charcoal (Cu/C) has been repeatedly employed as a cheap and relatively effective catalyst in CuAAC reactions [88,89]. While the occurrence of CuO and Cu_2_O particles is generally acknowledged in Cu/C obtained by calcination [103,104,105], recently Buckley et al. [106] identified unsupported gerhardite (Cu_2_(OH)_3_NO_3_) as the likely catalyst precursor in materials prepared by the Lipshutz′ low-temperature carbon impregnation method [107,108]. Upon exposure to CuAAC conditions, the Cu(II) salt was found to transform into the actual active form, deemed to be a polymeric Cu(I) acetylide. The latter could be recovered by filtration, along with unreacted gerhardite, but no recycling of the material was reported. In this context it is worth noting also that Decan and Scaiano provided a detailed picture of commercial Cu/C catalyst under *in operando* conditions [109]. The study, based on single molecule fluorescence microscopy for mapping CuAAC events, suggested that 90% of the charcoal particles in the sample were devoid of any active site. Moreover, for the remaining 10% ones the active sites were estimated to represent just a minute fraction (approximate 0.003%) of the total surface. These results might help to explain the scarce catalytic activity, reported in some investigations for Cu/C samples from commercial sources [106,110].

An on-purpose prepared Pd-Cu bimetallic catalytic system on charcoal was studied by Rossy et al. for one-pot sequential Sonogashira-click and click-Heck reactions [111]. Unfortunately, an attempt to recycle the material in the former reaction evidenced essentially complete loss of its catalytic activity.

D’Haullin et al. prepared ultra-small copper oxide nanoparticles on graphite by stirring a suspension of the powdered support in a solution of Cu(OAc)_2_ in MeOH, kept under H_2_ [112]. The catalysts, which consisted of CuO, Cu(OH)_2_ and minor amounts of Cu_2_O, displayed fair activity in three-component CuAAC reactions of halides and styrene oxide (Scheme 2a,b). Although no recycling of the material was apparently attempted, filtration of the reaction mixture through a Nylon membrane permitted the prompt removal of the insoluble system and left only trace amounts of copper in the filtrate (6 ppb).

**Scheme 3:**
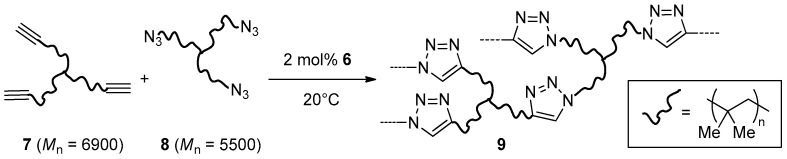
Click polymerization of alkyne and azide PIB trifunctional reactants.

Cu_2_O nanoparticles on graphene oxide (GO, **6**), were obtained by Nia et al. through high-temperature treatment in an Ar stream of GO impregnated with Cu(OAc)_2_ [110]. The material proved more active than commercial Cu/C and Cu_2_O in two-components CuAAC reactions (Scheme 1), but a relatively large catalyst loading (2 mol %) and extended reaction time (48 h at 40 °C) were still necessary for attaining good results. Moreover, recycling experiments in the benchmark reaction between benzyl azide and phenylacetylene in THF showed a marked decrease of the product yield already in the fourth run (99% → 55%). Interestingly, **6** was effective also in the bulk cross-linking of a mixture of alkyne and azide trifunctional macromolecular reactants **7** and **8**, based on star poly(isobutylene) (PIB) chains (Scheme 3). Although in these cases trapping of the copper nanocomposite inside the resulting resin **9** prevents its reuse, it is worth mentioning that a kinetic study of click-polymerizations promoted by **6** was published also [113].

**Scheme 4:**
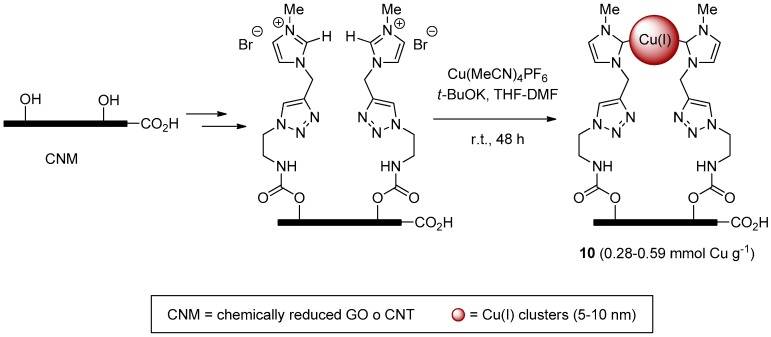
Preparation of Cu(I)-carbene clusters **10** on carbon nano-materials.

More recently, the same group reported highly dispersed copper clusters on carbon nano-tubes (CNT) and chemically-reduced graphene oxide (CRGO) [114]. These systems **10**, supposed to embed Cu(carbene)X or Cu-Cu fragments on the basis of literature precedents for Ag(I) analogs [115], were obtained by a stepwise procedure culminating (Scheme 4) in the decoration of the supports with triazole-imidazolium units and formation of Cu(I)-carbene clusters. Despite the not so much dissimilar copper content of CNT and CRGO-based materials **10**, the latter displayed a higher catalytic activity and better recycling profile than the former (respectively, 99% → 91% and 60% → 45% yield in 10 runs) in two-components CuAAC reactions (Scheme 1) and in the click-reticulation process mentioned above (Scheme 3).

Even in this case, the runs had to be carried for long time (24–72 h at 40 °C) and with substantial amounts of catalyst (2 mol %–8 mol %). In spite of this, copper concentration in the filtrate from the reaction mixture was reportedly too low for accurate quantification.

Another instance of copper oxide nanoparticles on GO was described by Reddy et al. [116]. The preparation of the supported system was achieved through a published procedure [117], whose key steps are the intercalation of Cu(II) between GO sheets and the subsequent exfoliation of the material, caused by nucleation and growth of CuO crystallites. TEM and XRD characterization of the nanocomposite confirmed the presence of CuO nanoparticles (15–50 nm) onto a graphene-like support. At variance with the examples discussed above and Cu_2_O on bare CRGO [118], the GO-CuO system proved highly active in water at 25 °C, even at 0.227 mol % loading. Under these conditions the addition of various benzyl or aryl azides to terminal alkynes (Scheme 1) proceeded in 10–20 min to give the corresponding triazoles in high yield (85%–96%). In addition to good dispersion of the nanocomposite, the result was attributed to hydrophobic phenomena at the carbon surface [119,120], related to the so called “Breslow effect” [121]. Filtration through a 0.2 μm PTFE membrane allowed the separation of GO-CuO and its reuse in five cycles, where some decrease of product yield was noted even with the adoption of progressively longer reaction time (approximate 96% → 90%). The effect was ascribed to leaching of CuO in the water phase, estimated to occur at a pace of around 0.6 wt % per cycle.

**Scheme 5:**
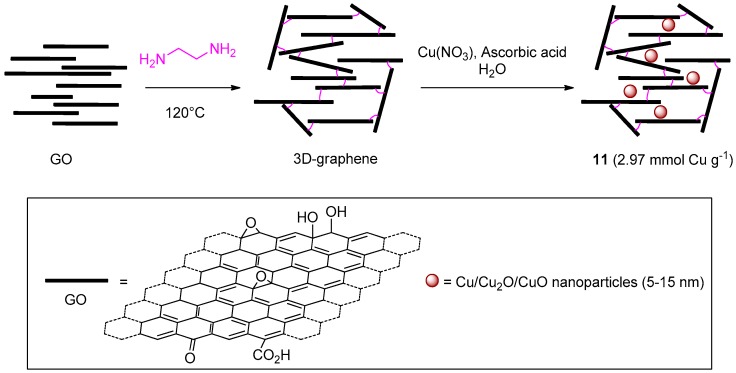
Preparation of 3D-graphene/CuNPs nanocomposite **11**.

A last recent entry in the list of GO-based materials for CuAAC comes from the work of Dabiri et al. (Scheme 5) [122]. In this study, GO was reacted with ethylenediamine with the aim of preparing a 3D graphene hydrogel that, in a subsequent step, was decorated with oxidized CuNPs, from the reduction of Cu(NO_3_)_2_, to give **11**. In a series of two- and three-components reactions in EtOH-H_2_O (1:1), the catalytic activity in CuAAC of the resulting system resembled that of other carbon-based materials noted above. In particular, relatively high loading and reaction temperature (5 mol % and 70 °C) respectively, had to be adopted for attaining good yields within 1–7 h (81%–99%). On the other hand, the 3D-graphene material displayed a better recyclability than most of the analogous systems, as witnessed by minimal reduction of product yield at fixed reaction time even after 10 consecutive cycles (99% → 97%).

### 2.2. Insoluble Synthetic Organic Polymers

**Scheme 6:**
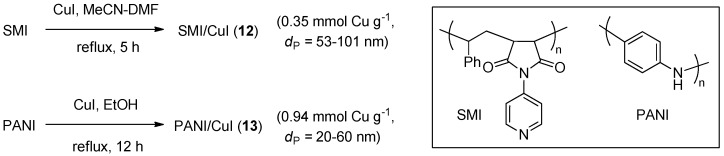
Preparation of CuI nanocrystals on SMI and PANI polymeric supports, **12** and **13**.

CuAAC catalytic systems on organic polymers, **12** and **13** (Scheme 6), were obtained recently by depositing nanoparticles of cuprous halides on a poly(styrene-*co*-maleic anhydride) imide (SMI) or polyaniline (PANI). The former support material, studied by Hashemi et al., was obtained by treating styrene-maleic anhydride copolymer with 4-aminopyridine under dehydrating conditions [123]. Subsequent refluxing with CuI nanoparticles, prepared by DMF precipitation of the cuprous salt from a MeCN solution, led **12**, that was shown by SEM to contain an uniform dispersion of copper halide crystals within the fiber-like polymeric matrix. In a similar approach, **13** was obtained by Saadat et al. by refluxing PANI and CuI in EtOH, under an inert atmosphere [124]. The catalytic activity of the materials was evaluated in three-component CuAAC reactions of activated halides or methyl iodide (Scheme 2a). The reactions were typically carried out in boiling water, with 1 mol % of **12** or, respectively, 5 mol % of **13**. Despite the potential solvolytic instability of the halide reactants under these conditions, fair to good yields were reported in both studies (71%–92% in 15–145 min). The scarce solubility of the respective support material in water, as well as in most organic solvents, allowed the recovery and reuse of the catalytic systems. Also from this point of view, the findings of the two investigations were quite similar, with some decrease of product yield (approximate 92% → 83% and 92% → 88%, respectively) and a moderate reduction of metal loading on the support (5% and 3%, respectively) after five consecutive runs.

**Scheme 7:**
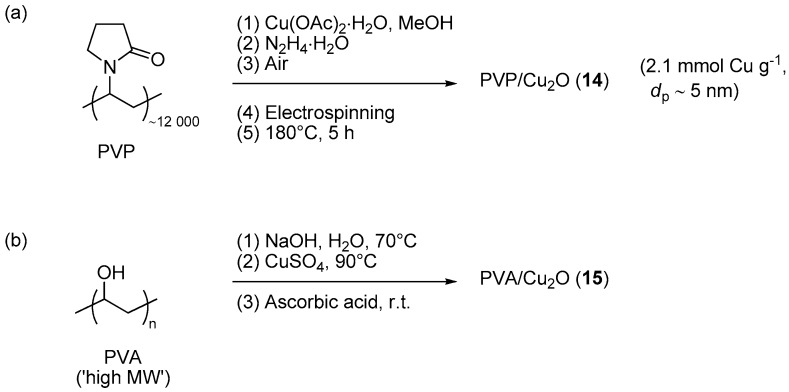
Preparation of PVP/Cu_2_O membrane **14** (**a**) and of PVA/Cu_2_O composite **15** (**b**).

Savva et al. investigated the use of electrospinning as a mean for obtaining Cu_2_O nanoparticles embedded into membranes made of cross-linked poly(*N*-vinyl pyrrolidone) (PVP) [125]. The preparation route (Scheme 7a) involved hydrazine reduction of a mixture of linear PVP and Cu(OAc)_2_, followed by electrospinning of the resulting colloidal solution on a target collector and final curing at 180 °C. With this procedure, initially formed Cu(0) clusters were probably oxidized to Cu_2_O and a material **14** was obtained that, contrarily to linear PVP, resulted insoluble in water. Interestingly, the curing stage did not cause dramatic variations of the morphology of the membrane, in particular for what it concerns the occurrence of cylindrical polymer fibrils supporting a uniform dispersion of metal oxide particles. The electrospun composite membrane **14** was effective in promoting the CuAAC addition of benzyl azide to phenylacetylene in 1,4-dioxane and in the presence of an equivalent amount of Et_3_N. However, a rather high metal loading (5 mol % Cu) was used in these experiments in order to obtain the triazole product in 87% yield after 2 h at 60 °C. Moreover, reuse of the material in three consecutive runs indicated a gradual loss of catalytic activity (87% → 77% yield), accompanied by aggregation phenomena of the polymer fibers.

The use of poly(vinyl alcohol) (PVA) for stabilizing Cu_2_O nanoparticles was explored by Hajipour et al. [97]. In this case, the preparation of the catalytic system (Scheme 7b) involved the initial formation of a gel from PVA and CuSO_4_ in alkaline NaOH solution, followed by reduction with sodium ascorbate, aging, filtration, and washing. Drying in air afforded a reddish product **15**, characterized by a broad UV band centered around 491 nm and an XRD pattern matching that of Cu_2_O. Interestingly, the choice of basic conditions (pH = 12) proved essential for the reduction to Cu(I) because the same step, carried out without the addition of NaOH, led to a material featuring the XRD diffractions of metallic copper. Screening of PVA-Cu_2_O in CuAAC involved reactions of preformed aromatic azides (Scheme 1), or benzyl azides formed in situ from the corresponding halides and NaN_3_ (Scheme 2a). In both cases the reactions were run in water with a low, yet unspecified, copper loading and in the presence of tetrabutylammonium bromide. Rather surprisingly, the latter proved beneficial even in the former scenario, i.e., where the action of a typical phase-transfer catalyst was expected to be marginal due to the lack of ionic reactants. Under the said conditions the CuAAC runs proceeded smoothly to give the expected triazoles in reasonable yield (68%–95% in 1–9 h). Moreover, the activity of the pristine catalyst was retained with the insoluble material recovered by decantation and washing, as confirmed by essentially constant results in the course of 4 reaction cycles (95% → 94% yield).

**Scheme 8:**
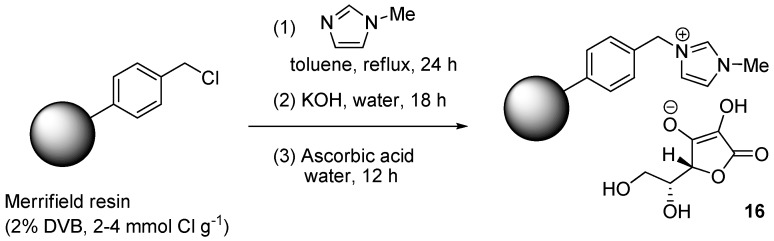
Preparation of resin-supported ascorbate **16**.

In an unusual approach, Patil et al. immobilized the ascorbate reducing agent on a polystyrene resin decorated with *N*-methylimidazolium ionic fragments [126]. The material **16** was obtained (Scheme 8) by reacting a Merrifield resin with *N*-methylimidazole, exchanging hydroxide ions for the chloride ones and then treating the basic form of the material with ascorbic acid. The supported reducing agent (16 mol %) was employed in three-components CuAAC reactions in water of aromatic diazonium tetrafluoroborates (Scheme 2c), implemented by making use of CuSO_4_ (4 mol %) as the soluble catalyst precursor. While this choice is expected to lead to progressive consumption of the ascorbate co-catalyst, TEM-EDS and XPS analyses of the resin recovered at the end of CuAAC runs suggested the presence of CuNPs on the material (probably CuO, approximate 0.52 mmol·g^−1^). At the same time, no metal was found in the filtrate by AAS, thus offering the possibility of recycling the (in situ) immobilized catalyst. This was effected with reasonable albeit incomplete efficiency, as confirmed by the observation of significant yield values through 6 consecutive reaction cycles (94% → 85%).

### 2.3. Metal-Organic Frameworks

**Scheme 9:**
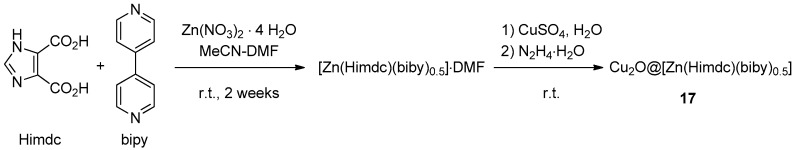
Preparation of Cu_2_O nanoparticles in 2D metal-organic framework, **17**.

Metal-organic frameworks (MOFs) represent an interesting class of hybrid organic-inorganic porous materials with tunable properties [127]. The preparation of Cu_2_O nanoparticles immobilized within the pores of (Zn(Himdc)(bipy)_0.5_) (Himdc = 4,5-imidazoledicarboxylate; bipy = 4,4′-bipyridine, Scheme 9) was achieved by Jayaramulu et al. through reduction with hydrazine hydrate of the CuSO_4_-impregnated MOF [128]. XRD, XPS, and TEM characterization of the resulting solid **17** showed the presence of Cu_2_O nanoparticles (mainly 2–3 nm in size and containing minor amounts of CuO), dispersed within the pores of the essentially intact Zn(II)-ligands framework. Two-components CuAAC catalytic runs (Scheme 1), in the presence of an equivalent amount of Et_3_N and 0.5 mol % of **17** in *t*-BuOH-H_2_O (2:1), afforded nearly quantitative yields after 7 h at 50 °C. The MOF-supported catalyst could be recovered by filtration, dissolution of the triazole product in CHCl_3_ and centrifugation, thus allowing the completion of three reaction cycles with reportedly unchanged activity.

Considering also that TEM, XRD, and CO_2_ adsorption measurements showed no significant changes with respect to the pristine material, these result led to the conclusion that pendant Himdc carboxylate groups in the MOF structure may have a positive role in stabilizing Cu_2_O nanoparticles and preventing their aggregation. Unfortunately, the lack of any information about the content of copper and MOF components in the crude triazoles (either Zn(II) or the organic ligands) makes it difficult to draw any conclusion about this interesting CuAAC system from the point of view of product contamination.

### 2.4. Polymers from Natural Sources

**Scheme 10:**
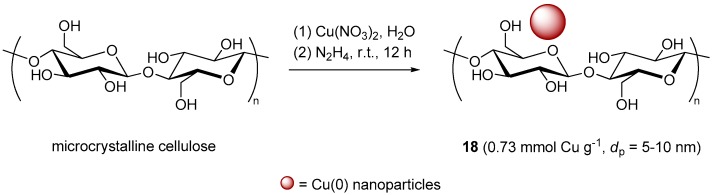
Preparation of Cu nanoparticles on cellulose, **18**.

With the specific goal of overcoming the modest yields in the CuAAC of some glycosyl azides with the standard CuSO_4_/sodium ascorbate system, Yu et al. endeavored to prepare CuNPs on cellulose (Scheme 10), by reduction of Cu(NO_3_)_2_ with hydrazine in the presence of the polymeric support [129]. Despite the fact that distinction between Cu(0) and Cu(I) is not always easy [130], the observation a XPS Cu 2p_3/2_ peak at 932.68 eV in the spectrum of the insoluble material **18** was taken as a proof of the occurrence of clusters containing metallic copper. Evaluation of the activity of **18** in the CuAAC reaction of phenylacetylene with acetylated *α*-l-arabinopyranosyl azide displayed a good activity at r.t. in several organic solvents. However, for the sake of better eco-compatibility water was eventually selected as the medium of choice, resulting in an optimized protocol that required the use of 10 mol % of supported catalyst at 60 °C for 12 h. Under these conditions, a range of protected glycosyl azides were coupled with different alkynes, to provide the corresponding triazoles in good isolated yields (84%–94%).

Because the cellulosic material and the triazole products were all poorly soluble in water, recovery of the catalyst required solvent extraction with AcOEt of the residue obtained by filtering the reaction mixture. Even if no apparent loss of supported system was noted at this stage, reuse of the material across five CuAAC cycles caused an evident decrease of product yield (93% → 84%). In consideration of the minute amounts of found copper into the filtrate (0.3–1.9 ppm), leaching phenomena were concluded to be irrelevant in this regard. On the contrary, the origin of the problem was tentatively related to oxidation phenomena, as suggested also by the observation of new peaks at 933.28 and 934.88 eV in the XPS spectrum of the spent catalyst, attributed to Cu(I) and Cu(II) species.

### 2.5. Oxides and Other Inorganic Supports

Although details of the preparation and structure are not entirely clear, Agalave et al. reported the use of CuI on bare neutral alumina as a cost-effective supported CuAAC catalyst [131]. The material was used in the reactions of activated halides (Scheme 2a) or a few preformed azides (Scheme 1), in water or DMF-water (4:1) with microwave (MW) or ultrasound (US) irradiation. The former conditions (MW) led to the fastest conversion rate and furnished good yields in 6–13 min, even in the case of triazole products with relatively complex structure (70%–98%). Reuse of the recovered catalyst in the course of eight reaction cycles displayed a progressive decrease of efficiency (approximate 98% → 89% yield), accompanied by some reduction of the copper content of the material (4.2 wt % → 3.8 wt %).

KIT-5 mesoporous silica modified with aminopropyl groups was employed by Mirsafaei et al. for supporting CuI nanoclusters [83]. In spite of the fact that the aminated support was simply stirred at r.t. in a solution of CuI in MeCN or DMF, XRD measurements identified the presence of crystalline CuI (Marshite structure, estimated size 12–40 nm) in the resulting materials. The usefulness of the supported catalyst in CuAAC was assessed in the three-component process with activated alkyl halides (Scheme 2a). Good yields were generally obtained in refluxing water, in some cases after very short reaction time (69%–95% in 15–150 min). In addition, the recovered material could be used in 6 consecutive cycles with just a minor decrease of product yield (approximate 96% → 91%) and an unspecified small variation of the copper content.

Reduction of Fehling solution with glucose in boiling water, in the presence of various insoluble materials (silica gel, hydroxyapatite, cellulose, basic alumina), was used by Gupta et al. for preparing supported Cu_2_O nanoparticles [132]. Amongst the systems examined, that based on silica gel showed maximum activity in the three-component reaction (Scheme 2a) between phenylacetylene, benzyl chloride, and sodium azide. The reaction was carried out with a relatively high catalyst loading (5 mol %), preferentially in water at r.t. Under these conditions very good yields (91%–97%) were obtained in reasonable time (2–8 h), also on switching to the use of less reactive alkyl halides (e.g., *n*-butyl chloride). Reuse of the catalyst resulted in only slight reduction of product yield in the course of 5 cycles (96% → 92%).

Albadi et al. employed a CuO/CeO_2_ nanocomposite (approximate 5 wt % Cu) for performing three-components reactions with activated bromides (Scheme 2a) and Amberlite-supported azide [133]. The latter was selected with the intent of reducing the environmental impact of the process and to avoid handling toxic NaN_3_, at least in the CuAAC step. Optimization of the conditions revealed that the rate of CuAAC was maximum in refluxing ethanol, where different triazole products were obtained in good yield within 45–120 min (88%–92%). Interestingly, the CuO-CeO_2_ nanocomposite and the resin were recovered together by filtration and the latter was restored in the azide form by stirring with aqueous NaN_3_. With this procedure, the attainment of nearly unchanged yields in the course of five reaction cycles (91% → 88%) required a slight increase of the reaction time, from 65 min to 75 min.

The same group prepared CuO/ZnO nanocomposites by co-precipitation of the metal nitrates with aqueous NaHCO_3_ [134]. The use of the material in three-component CuAAC reactions of benzyl halides (Scheme 2a) provided best results in boiling water and with a high loading of the catalyst (89%–92% yield in 15–65 min with approximate 17 mol % Cu). Also in this case, reuse of the recovered solid led to an evident decrease of catalytic activity after just five consecutive reaction cycles (92% yield in 20 min → 88% yield in 32 min).

Very recently, Rad et al. described the CuAAC preparation of β-hydroxyalkyltriazoles, containing carbazolemethyl side chains, by the use of copper-doped silica cuprous sulfate [135]. Contrarily to previous work by the same group [136], no recycling of the catalyst was reported in this example of application to the synthesis of bioactive compounds.

### 2.6. Magnetically Recoverable Systems

The use of nano-sized super-paramagnetic supports offers the opportunity of catalyst recovery by the use of an external magnet. In principle, this technique can be more advantageous than filtration or centrifugation, even at scale, thus justifying the interest towards the approach in last decade [137,138]. Hence, irrespective of the fact that many of these materials embed additional organic or inorganic components, recent advances with nano-structured copper catalysts that can benefit from such a specificity recovery mechanism are discusses separately in the present section.

**Scheme 11:**
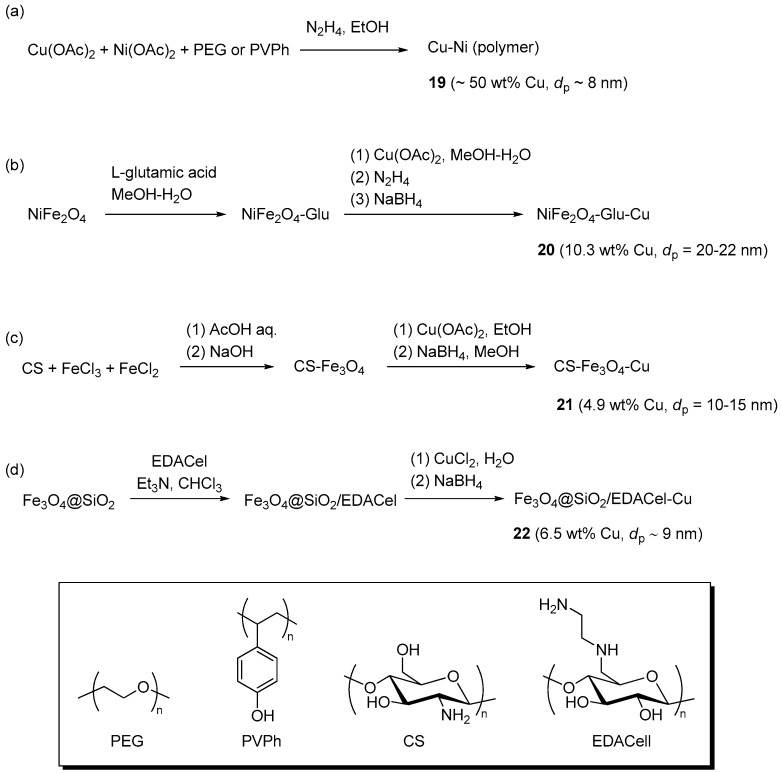
Preparation of CuNPs on super-paramagnetic supports with PEG or PVPh (**a**); l-glutamic acid (**b**); chitosan (**c**); and EDACell (**d**) stabilizing agents.

Magnetic Cu-Ni nano-alloy particles were obtained by Biswas et al. through reduction of nickel acetate and copper acetate with hydrazine [139]. The preparation (Scheme 11a) was carried out in the presence of PEG or poly(4-vinylphenol) (PVPh) as a polymer stabilizer, to give well dispersible organic-inorganic hybrid materials **19**. TEM and XPS analyses showed the presence of spherical alloy nanoparticles containing a Cu(I) surface layer and assembled in chain-like structures through the interaction of their magnetic dipoles. Benchmark runs (Scheme 1) between benzyl azide and phenylacetylene were carried out at r.t. in water or DMF, with an amount of nano-alloy corresponding to about 2 mol % of copper. Under these conditions, the PEG-containing catalyst **19** was found to be equally effective in both reaction media.

By contrast, the PVPh-stabilized one proved significantly more active in DMF than in water, a difference attributed to the collapsed state of the less polar PVPh coating in the latter solvent. In any case, with the proper choice of reaction conditions either catalytic system provided acceptable results in CuAAC reactions involving other low-molecular weight reactants (56%–82% yield), a propargylated imidazolium ionic liquid, or azide-terminated substrates containing a tripeptide or a poly(methyl methacrylate) chain. Recovery of the catalyst by magnetic separation allowed the use of **19** (PEG) in three consecutive benchmark runs, albeit with reduction of the triazole yield (96% → 87%). Because the XPS analysis of the recovered material showed some increase of Cu(I) peaks but not of those of Cu(II), this trend does not appear to be related to severe oxidation of the nano-alloy surface. On the contrary, it might be connected with particle aggregation phenomena, evidenced by TEM analysis of the spent catalyst.

A tris-metallic system, entailing Cu(0) nanoparticles on glutamate-coated NiFe_2_O_4_ (Scheme 11b), was prepared by Lu et al. by sequentially loading the super-paramagnetic core nano-sheets with the amino acid ligand and Cu(OAc)_2_, followed by reduction with N_2_H_4_ and NaBH_4_ [140]. The resulting material **20** was examined as a catalyst in multi-component CuAAC reactions in water, involving activated halides, epoxides, or aromatic boronic acids (Scheme 2a,b,d, respectively). In the presence of the composite catalyst (1 mol %–5 mol % Cu) the reaction proceeded in relatively short times at r.t. (1.5–8.5 h), allowing the preparation of a rather large library of triazole products in fair to excellent yields (50%–95%). Recovery with an external magnet and washing made it possible to reuse the supported catalyst in ten successive cycles, with moderate decrease of the product yield (95% → 88%).

CuAAC reactions promoted by Cu(0) nanoparticles on chitosan (CS)-magnetite have been described by Chetia et al. [141]. The nanocomposite was prepared by a three-step process (Scheme 11c), that began with the NaOH induced co-precipitation of CS, Fe(II), and Fe(III) from an AcOH solution. Characterization of the resulting material **21** by an array of techniques led to the conclusion that copper was present in the crystalline elemental form, as 10 nm spherical nanoparticles. Despite the fact that these dimensions do not appear much different from those estimated for the Fe_2_O_3_-CS support (10–15 nm), a uniform dispersion of copper on the surface of the nanocomposite was inferred at the SEM resolution level. Two-components CuAAC runs (Scheme 1) were preferentially carried out in CH_2_Cl_2_, with 1.5 mol % of the catalyst, to afford good to excellent yields in 12 h at r.t. (77%–96%). A high yield was recorded also in the reaction of dimethyl acetylenedicarboxylate with phenyl azide (96% in 12 h), from which it was concluded that **21** was effective even for internal alkynes. In this respect it should be noted, however, that acetylenedicarboxylate esters are known to undergo the thermal Huisgen 1,3-dipolar cycloaddition reaction at rate not dissimilar to that demonstrated in the study under exam, or even faster [142]. On this ground, the catalytic role of the nanocomposite material in the specific reaction of such an internal acetylene appears therefore far less than obvious. Magnetic separation of the catalyst beads allowed their recycling with just a moderate reduction of efficiency along four consecutive runs (96% → 91% yield). The occurrence of aggregation phenomena, as a possible cause of performance loss, and of catalytic activity in solution, due to leached specie, were examined and ruled out by TEM microscopy and through a standard “filtration experiment” (Maitlis′ test) [143], respectively.

An organic-inorganic hybrid material (Scheme 11d), consisting in a layer of ethylenediamine-functionalized cellulose (EDACell) adsorbed onto silica gel-coated Fe_3_O_4_ particles, was recently employed by Bhardwaj et al. as a novel support for CuAAC catalytic systems [144]. Reduction with NaBH_4_ of the material loaded with CuCl_2_ gave a dark solid **22** that, according to XRD and electron microscopy, contained metallic copper clusters uniformly dispersed on the surface of around one order of magnitude larger core-shell magnetic nanoparticles. The activity of the supported system was probed in the CuAAC reaction between acetylenes and aromatic azides, with the latter formed in situ by substitution with NaN_3_ on the product of diazotisation of the corresponding aniline in HCl-H_2_O (1:1) (Scheme 2c). An initial optimization stage demonstrated good performance at r.t. with as little as 0.25 mol % of the copper catalyst. Therefore, the same conditions were applied to the CuAAC of different substrates, to afford uniformly good results in relatively short reaction time (92%–95% yield in 3.5–5 h). Despite the use of a strongly acidic reaction medium, filtration tests did not reveal any significant catalytic activity in the solution. In addition, the copper content of the solid recovered by magnetic separation proved almost identical to that of the pristine material and its recycling led to almost unchanged results in the course of six consecutive runs (96% → 93% yield).

In addition to hybrid (organic-inorganic) magnetic catalytic systems above, some progress with purely inorganic materials were reported also. These examples, mentioned here for the sake of completeness, include the use of commercially available copper ferrite (CuFe_2_O_4_) and Fe_3_O_4_@TiO_2_/Cu_2_O nanocomposites [145,146].

### 2.7. Polymer-Stabilized Soluble Systems and Quasi-Homogeneous CuNPs

Leaf extracts from *Otostegia persica* and *Ginkgo biloba* were recently examined by Nasrollahzadeh et al. as green reducing agents in the preparation of unsupported CuNPs [147,148]. These systems showed reasonable catalytic activity and possibility of reuse in three-component CuAAC reactions. Similarly to other quasi-homogeneous nanostructured materials reported in the last two years, i.e., lanthanum-loaded CuO nanoparticles [149], porous copper [99], copper salts reduced in ethylene glycol [101], and Cu_2_O nanocubes [96], these systems do not appear however to involve any additional polymeric component. Therefore, according to the scope of this review they will not be discussed in further detail herein.

**Scheme 12:**
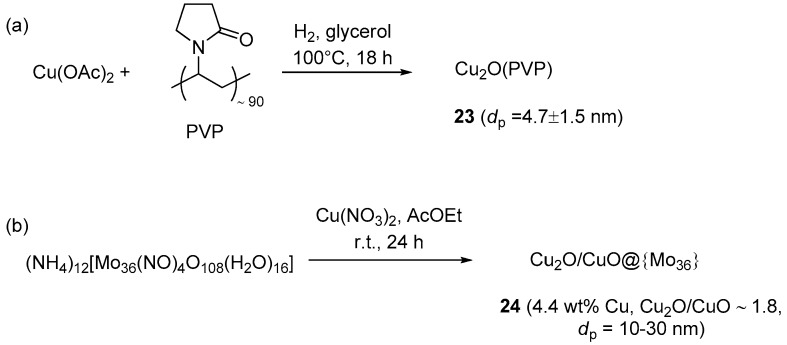
Preparation of soluble and quasi-homogeneous CuAAC catalysts **23** (**a**) and **24** (**b**).

On the contrary, Chahdoura et al. prepared polymer-stabilized Cu_2_O nanoparticles, by dihydrogen reduction of copper acetate in glycerol in the presence of PVP (Scheme 12a) [95]. As confirmed by TEM, XRD and IR analyses, the use of glycerol as the solvent, H_2_ as the reducing agent, and PVP as the stabilizer turned out to be all important for obtaining oxidation-stable Cu_2_O clusters devoid of Cu(0) or Cu(II) phases.

**Scheme 13:**
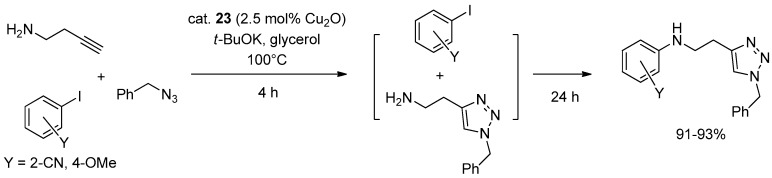
Tandem CuAAC-Ullmann three-component coupling promoted by **23**.

The synthesized nanoparticles **23** proved moderately active in two- and three-components CuAAC reactions (Scheme 1 and Scheme 2a). The catalytic runs were carried out with 2.5 mol % Cu_2_O at 100 °C for 2 h to give an array of different triazoles in high yield (91%–98%) and with complete 1,4-regioselectivity. In this regard it is worth noting that, even if the thermal reaction proceeded appreciably in neat glycerol at 100 °C (40% yield in 2 h), as expected both regioisomeric products **1** and **2** (see Scheme 1) formed in substantial amounts (4:1 ratio) in the absence of the copper catalyst. Combination with Ullmann chemistry was also feasible and provided a tandem process for preparing arylaminoethyl-functionalized triazoles (Scheme 13). The separation of the products in standard CuAAC runs was effected by extraction with CH_2_Cl_2_ or pentane of the catalyst-containing glycerol layer. Afterwards, the glycerol phase was heated under vacuum for removing the volatiles and employed in a new experiment. With this procedure ten cycles of the reaction between benzyl azide and phenylacetylene were carried out, obtaining the corresponding triazole product in nearly constant yield (94%–97%).

Polyoxometalates (POMs) and heteropolyacids (HPAs) are ionic compounds containing cations and large polyanion clusters made, e.g., by the condensation of MOx polyhedra [150,151]. Besides having catalytic activity on their own, these materials find use as supports for other catalytic systems and for the stabilization of metal nanoparticles. An example in this latter sense was reported recently by Amini et al. (Scheme 12b), who prepared Cu_2_O/CuO nanoparticles on a molybdenum POM by treating a suspension of (NH_4_)_12_(Mo_36_(NO)_4_O_108_(H_2_O)_16_) in AcOEt with Cu(NO_3_)_2_ [152]. With this procedure, the occurrence of the Cu(I) compound in the resulting material **24** was supposed to derive from direct reduction of the Cu(II) salt at the POM surface. Three-components CuAAC runs of activate halides (Scheme 2a) were typically performed in water, where the resulting nanocomposite was soluble. In the event, this permitted the recovery of the catalytic system in the aqueous phase, by filtering off the insoluble triazole product at the end of the experiment. Despite the rather high catalyst loading and reaction temperature adopted in these runs (5 mol % and 70 °C, respectively), recycling of the POM solution led, however, to rapid degradation of catalytic performance already after a few reaction cycles (approximate 88% → 44% yield in three runs).

## 3. Discrete Cu(I/II) Complexes

Well-defined Cu(I) salts and complexes with nitrogen, phosphorous, oxygen and carbon-based ligands have played a prominent role in the development of new effective systems for CuAAC [64,153]. Because properly designed ligands, firmly anchored onto an insoluble support, might prevent metal ions to leach from the material, it is not surprising that immobilized catalytic systems of this kind, as well as analogous Cu(II) pre-catalysts, were actively investigated [88,89].

### 3.1. Cross-Linked Synthetic Organic Polymer Supports

After the introduction in 2006 of CuI on Amberlyst A-21 by Girard et al. [154], this supported Cu(I) system has been successfully employed as a recoverable catalyst in CuAAC reactions carried out under batch or continuous-flow conditions (for a discussion of previous work, see refs. [88,89]). Occasional observations of C-5 iodinated triazole by-products in the cycloaddition process catalyzed by A-21/CuI led the same group to investigate the possible causes of such a side-reaction [155]. After examining various factors, potential mistakes were identified in the preparation of the supported catalyst and in the conduct of CuAAC runs, which result in the obtainment of substantial amounts of iodinated triazoles (up to 12 mol %). In turn, this allowed to establish optimized conditions, whose adoption is expected to reduce the formation of by-products below the 0.5 mol %–1 mol % level.

**Scheme 14:**
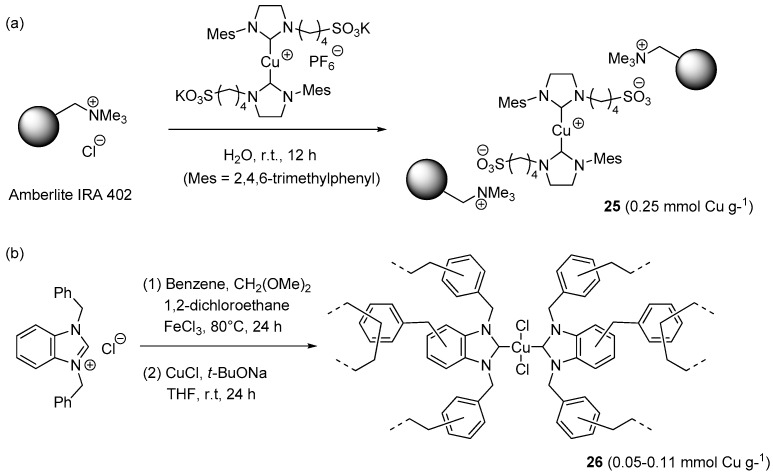
Preparation of NHC-Cu(I/II) complexes on insoluble organic supports, **25** (**a**) and **26** (**b**).

A ionically immobilized *N*-heterocyclic carbene (NHC)-Cu(I) catalyst **25** (Scheme 14a) was prepared by Velazquez et al. by suspending the gel-type, quaternary ammonium resin Amberlyst IRA-402 in an aqueous solution of the metal complex [67]. After washing with water, the loaded resin (2 mol % of Cu) was tested in three-component CuAAC reaction between benzyl bromide, sodium azide, and phenylacetylene in water (Scheme 2a). Possibly due to poor solubility of the reactants in water, and thence hindered access to the catalytic sites, mechanical stirring resulting ineffective under these conditions. The problem was circumvented by resorting to ultrasound irradiation, which permitted to attain full conversion in five hours at r.t. Remarkably, the same conditions worked fine also for the rarely investigated three-component reaction involving acetylene as the dipolarophile partner. Recycling of the resin was studied in the former process and involved extraction of the product with CH_2_Cl_2_, removal of the water phase by syringe, and subsequent addition of fresh reactants and solvent. By carrying out all the steps under an argon atmosphere, four consecutive runs could be performed, albeit with an appreciable reduction of product yield (99% → 75%).

A further attempt to reuse the supported complex was reported to lead to a dramatic, yet unspecified, further decrease. Although some details of the measurements are not entirely clear, XRF analysis suggested that leaching of Cu(I) species into the solution might be the cause of the problem.

Another instance of carbene-stabilized CuAAC catalyst on a resin support was reported very recently by Jia et al. [156]. The first step of the preparation route (Scheme 14b) consisted in the covalent immobilization of 1,3-dibenzylbenzimidazolium chloride into a hyper-crosslinked matrix, obtained by “knitting” the ligand precursor with benzene, 1,2-dichloroethane, and methylal under Friedel-Crafts conditions. Loading the resulting, highly porous insoluble material with different amounts of CuCl and *t*-BuONa led to the corresponding supported metal-carbene complexes **26**. Despite the fact that XPS analysis revealed the presence of Cu(II), instead of Cu(I), all the resins could catalyze the three-components CuAAC reaction of benzyl halides in EtOH (Scheme 2a). Best results were provided by the material with the largest metal content that, at the relatively low loading of 0.45 mol % Cu, afforded the expected triazole products in good to excellent yields within 8 h at 80 °C (61%–99%). Separation of the resin by centrifugation made it possible to use the supported catalyst in six consecutive cycles with essentially unchanged results (98% → 96% yield).

**Figure 3:**
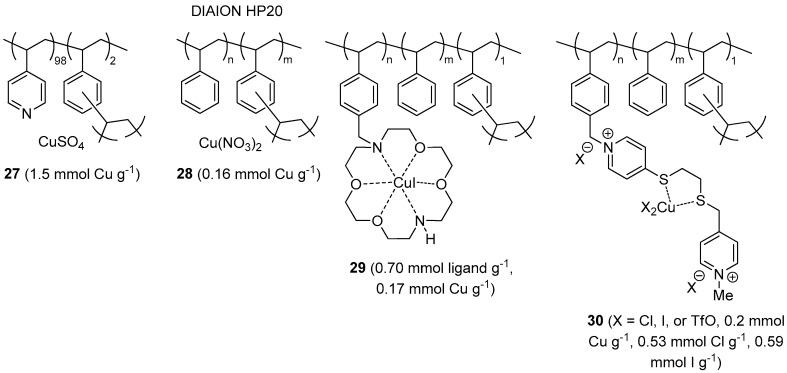
CuAAC catalysts and pre-catalysts **27**–**30**, on insoluble organic supports from commercial resins.

Commercial poly(4-vinylpyridine-*co*-divinylbenzene) was employed by Zarchi and Nazem for preparing the CuAAC pre-catalyst **27** (Figure 3), through absorption of aqueous CuSO_4_ on the gel-type resin [157]. After in situ reduction of the blue polymer with sodium ascorbate, the material was tested as a catalyst in three-component CuAAC of halides (Scheme 2a). Following an initial solvent screening, the reactions were performed in biphasic *t*-BuOH-H_2_O (1:1) medium to give various triazole products in fair to excellent isolated yields and short reaction time (69%–96% in 30–135 min). In this respect it must be noted, however, that heating to 70 °C was required and, more important, a higher than stoichiometric catalyst amounts was applied (225 mol % Cu, on the basis of published data). Even though the polymeric material could be used in six reaction cycles with slight reduction of the triazole yield (91% → 87%), its catalytic performance appears therefore somewhat less impressive than for many other recoverable CuAAC systems reported to date.

Following their seminal paper on CuAAC with copper catalysts on DIAION resins [158,159], more recently Kitamura et al. investigated the use of the highly porous styrene-divinylbenzene (DVB) material **28**, under solventless conditions [160]. Two-component catalytic runs (Scheme 1) were carried out at r.t. under Ar, with 1 mol % of the catalyst and the addition of substoichiometric amounts of Et_3_N to the neat reactants. Under these conditions, CuAAC between different azides and alkynes led to largely variable product yields after 4 h (47%–100%). The observation that worst results were obtained with dipolarophiles devoid of Lewis-basic heteroatoms (N, O) in the proximity of the C-C triple bond, led the Authors to conclude that neighboring-group participation may play an important role in the system under exam. Due to the fact that many triazoles are solid, isolation of the product required dilution with water and AcOEt before removal of the polymeric material by filtration. At variance with previous results with **28** in toluene [159], reuse of the resin under neat conditions showed a marked decrease of activity over four cycles (100% → 27% triazole yield). As expected, given the lack of strongly coordinating groups in HP-20, the origin of the problem was traced back to metal leaching which was indeed found to occur to a significant extent (19%) even in a single run. Moreover, assay of the separated liquid layers from product isolation (vide supra) revealed more than twelve-fold higher metal concentration in the AcOEt phase than in the aqueous one. Although copper contamination in the crude triazoles was not explicitly reported, an estimation of around 400 ppm (dry weight basis) can be made on the basis of published data.

A Cu(I) chelate catalyst **29**, immobilized on polystyrene resin, was described by Movassagh and Rezaei [161]. The supported complex was obtained by alkylating the diazacrown macrocyclic ether Kryptofix^®^ 22 with a Merrifield resin and then charging the resulting polymeric material with CuI in refluxing EtOH. Perhaps because of limited solubility of the copper salt in EtOH or scarce compatibility of the polystyrene backbone with the protic medium, the metal loading attained in the last step was considerably lower than the cryptand content of the resin. Nevertheless, **29** displayed appreciable activity in two- and three-components CuAAC reactions, especially when water was used as the reaction medium. Under air and at 0.3 mol % catalyst loading, several azides were added to acetylenic substrates (Scheme 1) to provide the corresponding triazole products in good to excellent yield after 10 h at r.t. (78%–99%). Analogous three-component CuAAC runs (Scheme 2a) with alkyl chlorides, bromides, or iodides and 0.6 mol % of supported complex were slower (56%–99% yield in 15–21 h at r.t.). Given also the structure of some of the halides used in the screening (e.g., cyclohexyl bromide and 1-adamantyl bromide), these variations are likely to reflect more the difficulty of the latter to be converted into the corresponding azide intermediate than actual differences in catalyst performance under alternative reaction conditions. Recovery of the supported system by filtration, washing, and drying with an air stream made it possible four consecutive reaction cycles, with a drop of product yield more evident under three-component conditions (99% → 71%) than under two-components ones (approximate 99% → 87%).

In a multi-step solid-phase synthesis approach, Tavassoli et al. pursued the preparation on Merrifield resin of a dicationic ligand provided with 1,2-dithioether chelating site [162]. Stirring of the material with Cu(OTf)_2_ in MeOH led to the corresponding Cu(II) complex **30**, characterized *inter alia* by cyclic voltammetry on glassy carbon electrode. Notwithstanding the presence of iodide in the material, addition of sodium ascorbate turned out to be essential for obtaining a catalytically active system for CuAAC.

In the presence of the reducing agent and with 0.2 mol % of **30** in PEG_400_ at 65 °C, quite fast reaction kinetics (87%–99% yield in 10–25 min) were observed in three-components CuAAC runs with mono-functional activated bromides (Scheme 2a). By contrast, somewhat reduced yields were recorded in the reaction of bis(bromomethyl)benzene starting materials, even after longer reaction time (68%–88% in 45–60 min). Despite the observation of some copper leaching in the first run (<2%), the supported system displayed a reasonable recyclability, with just a modest drop of product yield after seven consecutive cycles (approximate 99% → 94%).

**Scheme 15:**
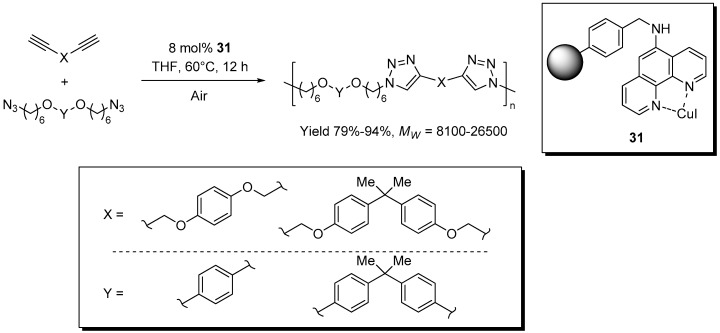
Click-polymerization of di-functional monomers in the presence of **31**.

A 1,10-phenanthroline-CuI catalytic system on Merrifield resin, **31**, was reported by Wu et al. for the click-polymerization of di-functional azide and alkyne reagents (Scheme 15) [163]. Similarly to previous work from the same group [79], the solubility in THF of the resulting linear polymers allowed the separation of the supported catalyst and the obtainment of polytriazole materials containing relatively low amounts of copper (190–410 ppm). At variance with the precedent study, however, in this case the process was found to proceed only in the presence of air and no recycling of the supported catalyst was attempted.

**Scheme 16:**
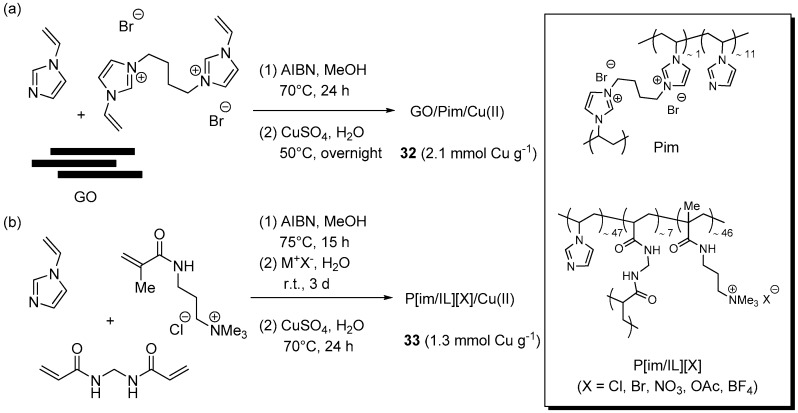
Preparation of supported Cu(I/II) complex **32** (**a**) and **33** (**b**) onto resins obtained by radical copolymerization and plausible structures of Pim and P(im/IL)(X).

In addition to the systems above, obtained from commercially available resins, examples were described also of materials prepared by radical copolymerization of functional monomers. For the purpose of increasing the loading capacity of GO and its effectiveness in retaining copper ions, Pourjavadi et al. resorted to the entrapment of the carbonaceous support into a cross-linked poly(vinyl imidazole) matrix (Pim) [164]. With this aim, *N*-vinylimidazole and the corresponding 1,4-bis-(imidazolium)butane di-functional monomer were radically polymerized in solution, in the presence of GO as a filler (Scheme 16a). Interestingly, while SEM examination of Pim devoid of GO showed a smooth surface, the nanocomposite was found to possess a corrugated morphology, attributed to the embedded GO sheets. Soaking the material in aqueous CuSO_4_ led to load a considerable amount of metal salt, corresponding to about one half of the imidazole units content estimated for the material. A preliminary optimization stage for the three-component CuAAC reaction of phenylacetylene, benzyl bromide, and NaN_3_ suggested the use of 1 mol % of the supported Cu(II) complex **32** and 10 mol % of sodium ascorbate, in water at 50 °C.

When the same experimental set-up was applied to the CuAAC of other terminal alkynes with benzyl or alkyl chlorides or bromides (Scheme 2a), good yields were uniformly recorded in 0.5–3.5 h (80%–96%). Remarkably, the reaction was found to proceed even with as little as 0.002 mol % of catalysts, but in this case a significantly longer reaction time was required for completion (20 h). Examination of leaching-related issues in the study under exam entailed a standard filtration test and the quantification of copper in the filtrate by ICP-OES. As desirable for a highly stable supported system, no evidence for catalytic activity in solution was gained by the former experiment, while in the latter the metal concentration was found to be below the detection limit. Therefore, the moderate reduction in product yield and TOF, observed in the course of eight recycle runs (approximate 99% → 92% and 195 → 140 h^−1^, respectively), was tentatively ascribed to catalyst loss during washing and recovery procedures.

The radical copolymerization of *N*-vinylimidazole with 3-methacrylamidopropyl trimethyl ammonium chloride and *N,N′*-methylenebisacrylamide (Scheme 16b) was employed by Pourjavadi et al. for preparing the cross-linked P[im/IL][X] material, provided with imidazole units for copper complexation and ionic trimethylammonium groups for tuning the catalytic properties by variation of the counter-ion X [165]. In fact, before charging with CuSO_4_, the resin was subjected to ion-exchange for the purpose of replacing chloride with other anions. Even though the result might look counterintuitive, because of the presence of a large excess of azide ions, when the different Cu(II)-loaded resins **33** were employed in three-component CuAAC runs (Scheme 2a) the activity of the supported systems was found to depend on the nature of the counter-ion in the order: Cl^−^ > AcO^−^ > Br^−^ > BF_4_^−^ > NO_3_^−^. Therefore, the chloride form **33** (X = Cl) was selected for being examined in more detail. This resulted in an optimized procedure that, with the use of 0.1 mol % of the supported system and 10 mol % of sodium ascorbate in *t*-BuOH-H_2_O (1:3) at 55 °C, provided high yields of triazole products in 1.5–6 h (85%–99%). Remarkably, when the catalyst amount was lowered to 0.0013 mol % (130 mol ppm) a respectable 91% yield was still obtained after 18 h (TOF = 3889 h^−1^). Moreover, under standard conditions the reuse of the material recovered by centrifugation allowed to perform 12 reaction cycles, without any appreciable reduction of product yield and turnover frequency.

**Scheme 17:**
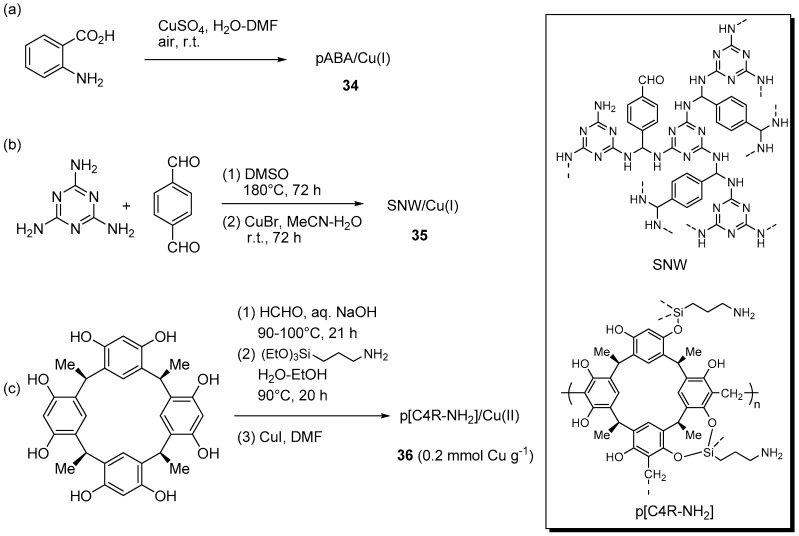
Preparation of supported Cu(I/II) complexes **34** (**a**), **35** (**b**), and **36** (**c**) onto resins obtained by oxidation or condensation routes and plausible structures of SNW and p[C4R-NH_2_].

A few resins and other insoluble polymers for the immobilization of CuAAC catalytic systems were obtained also by condensation or oxidative routes. For instance, an original approach to the preparation of a polymer-supported Cu(I) systems led Ul Islam et al. to realize polymer formation and copper immobilization at once, by addition of CuSO_4_ in DMF to 2-aminobenzoic acid in water, under open air (Scheme 17a) [166]. At SEM magnification, the resulting green precipitate **34** appeared to consist of robust fibers, devoid of metal particles down to the TEM resolution level. Therefore, the inference was made that the material was a complex between poly(2-aminobenzoic acid) (pABA), from the oxidative polymerization of the amino acid precursor, and Cu(I), from the partial reduction of the copper salt. The conclusion was strengthened by Raman, XPS and XRD analyses, with the latter additionally showing the crystalline nature of the organic polymer. Despite the low surface area (7.8 m^2^·g^−1^), the solid proved active in the benchmark addition of benzyl azide to phenylacetylene, reportedly carried out with 0.01 mol % Cu in solvents of different polarity. As revealed by kinetic measurements, the addition of Et_3_N (1 equiv. with respect to the reactants) was essential in order to improve the reaction rate and to avoid the occurrence of an initial induction period. Because H_2_O-Et_3_N led to best results, this medium was selected in the subsequent investigation of substrate scope, that led to the attainment of good to excellent yields at r.t. (84%–98% in 3–7 h) under both two-components (Scheme 1) and three-component reaction conditions (Scheme 2a). Although limited details of the procedure are provided, filtration of the reaction mixture and extraction of the triazole product with EtOAc allowed the recovery of the supported catalyst and its reuse.

This cause to some reduction of product yield across five cycles of a benchmark reaction (98% → 83%), explained in terms of catalyst losses during the washing stage. In this context, it is worth of note that, while ICP-MS analysis ruled out significant metal leaching in the filtrate, TEM examination of the catalyst after the first cycle showed the formation of rather large CuNPs (10–40 nm). Because the specific activity of the latter is essentially unknown, it cannot be excluded that some kind of sintering, e.g., by clustering of Cu(I) species or over-reduction, might contribute to the observed deterioration of catalyst performance.

The condensation of melamine with terephthalaldehyde allowed Taskin et al. to prepare a microporous Schiff-base network polymer (SNW) as a rigid and insoluble yellow powder (Scheme 17b) [167]. Solid-state CP-MASS, ^13^C-NMR and FT-IR characterization of the material identified the presence of aminal units, as well as residual functional groups of the starting compounds. This evidence prompted the Authors to depict the structure of the network as containing the diaminomethyl structural motif (-CH(NH-)_2_) instead of the expected Schiff base (-CH=N-) one. Prolonged stirring with CuBr in MeCN afforded the corresponding Cu(I) loaded material **35** that, similarly to the bare support, was found to possess a rather large micropore surface area (306 m^2^·g^−1^ and 548 m^2^·g^−1^, respectively). Testing of the immobilized catalytic system in two-components CuAAC reactions (Scheme 1), at unspecified loading, evidenced a relatively fast process in MeCN at r.t., with formation of the expected triazoles in fair to good yields (61%–98% in 90–300 min). This proved true even for a substrate, like propargyl acrylate, that can face selectivity problems in the Huisgen cycloaddition reaction [168]. Recycling of the material recovered by filtration displayed sustained catalytic activity in the course of 7 runs (98% → 94% yield), while AAS analysis of the filtrate from the reaction mixture could not detect any copper down to the ppb level.

A more complex system was reported by Mouradzadegun and Mostafavi, who immobilized CuI on a 3D-network polymer made by polycondensation of calix [4] resorcinarene and formaldehyde (Scheme 17c) [169]. With this aim, the resin was initially modified by the introduction of aminopropyl chains and then loaded with a solution of the cuprous salt in DMF to give **36**. The system was tested in the three-component CuAAC reaction of halides in water (Scheme 2a). Even though the catalyst (5 mol %) displayed reasonable activity at room temperature, reflux conditions were ultimately selected as they could provide good yields (80%–97%) within 5–30 min. Recovery of the resin by filtration and solvent washing permitted to confirm its potential reuse, with just a slight reduction of product yield in the course of 5 cycles (97% → 94%). At the same time, AAS analysis of the filtrate evidenced a not specified “trace amount” of copper ions.

**Scheme 18:**
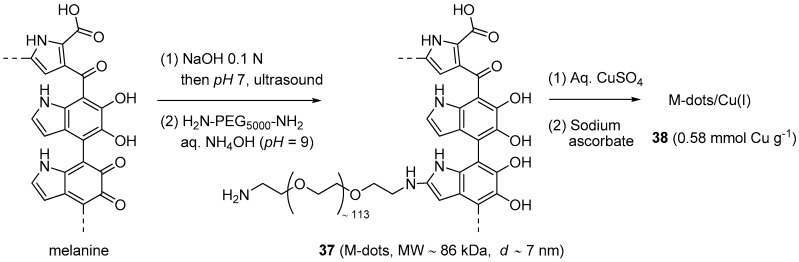
Preparation of the M-dots/Cu(I) nanoparticles and likely structure of M-dots (Note: The formula shown for the latter is the dihydroxyindole tautomer of the dihydroindolequinone one depicted in the original publication [171]).

After introducing PEG-modified melanin as a platform for multimodality imaging [170], Sun et al. explored its use as a nanosized support for CuAAC catalytic systems [171]. The preparation of the material (Scheme 18) began with the dissolution of synthetic melanine granules into NaOH, followed by US-assisted re-precipitation of the polymer on lowering the pH. After this step, aimed to disentangle the macromolecular chains, the resulting water-insoluble melanine nanoparticles were subjected to chemical modification with NH_2_-PEG_5000_-NH_2_ [170]. This second stage afforded nanoparticles **37** (M-dots) that, probably thanks to their surface charge (−2.2 mV zeta potential), resulted well dispersible in water but could be recovered by centrifugal separation through a 30 kDa cutoff filter.

After loading with an aqueous solution of CuSO_4_ and reducing with sodium ascorbate, the same technique was applied for the purification of the corresponding supported Cu(I) complex **38**. Characterization of the material by ICP-MS revealed that, on the average, each M-dot particle was loaded with 50 ± 2 copper centers, whose +I oxidation state was confirmed by XPS. Specific tests on the M-dots/Cu(I) system evidenced just a modest metal leaching in phosphate PBS buffer at pH = 7.4 and no toxicity against two cellular lines. In addition, M-dots displayed the ability to quench the production of hydroxyl radicals in the Cu(II)-ascorbate reactive system, thus circumventing one of the main issues when this chemistry is applied for bio-conjugation purposes. The appealing features of **38** prompted its study in CuAAC reactions between low molecular weight reactants (Scheme 1) and for the click-derivatisation of biomolecules. In the former scenario, very good product yields (90%–95%) were obtained in 30 min at r.t., by stirring the azide and alkyne reactants in PBS buffer in the presence of 0.1 mol % of the copper catalyst. Similar, albeit slightly worse results (67%–90% yield) were obtained in the corresponding three-component process with halides in PBS-DMSO (Scheme 2a). Reusability of the catalytic system was studied in the reaction of phenylacetylene and a tetra(ethylene glycol)-derived azide. With this choice, **38** could be separated by centrifugal filtration from the solution containing the triazole product, without leaving behind measurable amounts of copper. The subsequent direct reuse of the recovered catalyst, i.e., without adding any reducing agent, afford then reasonably constant results in the course of seven reaction cycles (88% → 82% yield).

In a second set of applications, **38** was used for click-modifying biotin and AE105 or RGDyK polypeptide derivatives, provided with either alkyne or azide side chains. Moreover, the same protocol could be applied to the fluorescent tagging of a 3’-alkyne DNA strand with the cyanine 5.5-azide dye, in this case using a 10 kDa cut-off centrifugal filter for separating the polynucleotide product from **38**. In a final noteworthy example, the in situ imaging of the integrin α_V_β_3_ receptor in human glioblastoma U87MG cells was realized. In this case, the procedure involved preliminary labelling the receptor sites with RGD-alkyne, followed by exposure of the whole cells to Cyanine 5.5-azide dye and **38**. As for the other biomolecules mentioned above, the possibility of using the melanine-based Cu(I) catalytic system, without the need of any added ascorbate, was undoubtedly a major advantage in order to prevent unwanted side reactions in the CuAAC process.

**Scheme 19:**
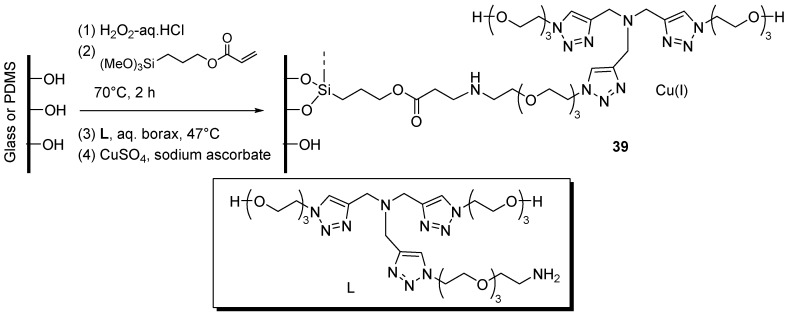
Immobilization of tris(triazolylmethyl)amine ligand/Cu(I) inside a glass-PDMS chip reactor **39**.

As anticipated in the Introduction, the necessity of handling potentially explosive azides under safe conditions prompted several studies on continuous-flow CuAAC [31]. In this context, the combination of flow-chemistry with another enabling technology [172], viz. the use of supported catalytic systems, stands as one of the most promising trends of current efforts towards the improvement of the reaction. Accordingly, several examples of in continuo CuAAC reactions have appeared in the literature, most of which make use of copper tubular reactors or supported systems in meso-sized fluidic devices [32]. Given the interest of fast CuAAC reactions on the pico-mole scale as a tool for, e.g., identifying enzyme inhibitors [173,174], Li et al. explored the preparation of glass-poly(dimethylsiloxane) (PDMS) chip micro-reactors containing immobilized tris(triazolylmethyl)-amine-Cu(I) units **39** (Scheme 19) [175]. The step by step progress of the fabrication sequence was verified by XPS analyses on test wafers, while radiochemical assay with ^64^CuSO_4_/sodium ascorbate allowed to quantify the capacity of the device in retaining Cu(I) (1136 ± 272 nmol or 81 ± 20 nmol·cm^−2^). Testing of the microfluidic reactor in two-components CuAAC (Scheme 1) was performed in a semi-batch fashion, by filling the channels with the reactants in ammonium acetate buffer and then allowing the reaction to proceed for some time (15–30 min) at 37 °C under stopped-flow conditions. Application of the procedure to the CuAAC coupling between the azide-modified fluorescent dye Flu568-N_3_ and propargylamine led to product yields that, as expected, increased at larger incubation times (e.g., 83% after 50 min vs. 55% after 15 min). Interestingly, the protocol was effective for performing also the click reaction between the azide-containing cyclic penta-peptide cyclo(RGDfK)-N_3_ and the functionalized dye Flu568-acetylene, with better results in the flow device than under analogous batch conditions (75% yield vs. 54% yield with 60 mol % of catalyst). Control experiments showed good reproducibility between different reactors and confirmed that the catalytic activity of non-specifically bound Cu(I), as found in bare chips, was just a fraction of that observed in the ligand-functionalized devices. The latter could be used 4–6 times before an appreciable reduction of product yield ensued. After that, full “regeneration” could be achieved by reloading the channels with CuSO_4_/sodium ascorbate. Arguably, while this observation points to a good stability of the supported ligand units, it evidences also possible leaching phenomena, whose extent was not quantified, in the product-containing liquid phase.

### 3.2. Polymeric Organic Supports from Natural Sources

As noted already in Section 2.6, chitosan, the biodegradable polymer obtained by deacetylation of chitin, is still attracting much interest as catalyst support in view of its low cost, inoffensive nature, and prompt adsorption of metal ions by surface hydroxy and amino groups. After early contributions by Chtchigrovsky et al. [176], the use in CuAAC of CS-Cu(I/II) complexes was examined recently by Nasir Baig et al. and by Anil Kumar et al. [177,178]. In both studies the supported polymeric catalysts were obtained by suspending CS in the solution of a proper Cu(I/II) salt, followed by filtration or centrifugation, washing, and drying of the insoluble material. Characterization of the solid indicated an appreciable copper content (5.1 wt % by ICP-AES), but no signals pertinent to crystalline forms were observed by XRD. The former group tested CS-CuSO_4_, at low catalyst loading (0.4 mol % of Cu) in water, in CuAAC between alkyl azides and aliphatic or aromatic alkynes (Scheme 1). On the contrary, the latter focused on the three-component CuAAC reaction between aromatic boronic acids, sodium azide, and aromatic alkynes (Scheme 2d). Also in this case water proved to be the solvent of choice, but the experiments involved the use of considerably larger amounts of catalyst (typically 10 mol % of CS-CuSO_4_). Under the said conditions good to excellent yields (71%–99%) were attained in both studies in 4–12 h at r.t. The CS-supported catalyst could be recovered by decantation and reused, albeit with variable degrees of success depending upon the specific reaction: With preformed azides, essentially quantitative yields were obtained in five initial runs and constant, yet reduced values (28%), were recorded in five additional reaction cycles carried out at five-fold larger S/C ratio. On the contrary, with boronic acids an appreciable reduction of yield was observed in the initial 5 cycles (90% → 79%), which appears to mirror quite closely the progressively larger losses of insoluble material along the reaction series (approximate 18% of the initial amount after the 5th reaction cycle).

Cuttlebone, the rigid structural component of the cuttlefish body, is an hybrid material composed of calcium carbonate (aragonite form), proteins, and β-chitin [179]. In view of the complexing/reducing properties of the latter component, Ghodsinia et al. explored the use of cuttlebone as a support for CuAAC catalytic systems [180]. Because XPS analysis of the solid obtained by treating powdered cuttlebone with CuCl_2_ in water confirmed the presence of copper in the Cu(I)/Cu(II) mixed oxidation state, the material was tested directly in the three-components CuAAC reaction of benzyl bromide and phenylacetylene. Strangely enough, bare cuttlebone displayed some catalytic activity itself but, as expected, the reaction rate in the presence of metal-loaded material proved significantly higher. Best results were attained by using 8.5 mol % of the supported copper catalyst in water at 40 °C, conditions under which the CuAAC multi-component reaction of other reactive bromides led to good triazole yields (72%–95%) within 5–80 min. Recovery by filtration and washing with different solvents allowed the reuse of the catalytic material in seven reaction cycles. This led to observe some decrease of substrate conversion and product yield (100% → 90% and 95% → 85%, respectively) and the reduction of the metal loading on the cuttlebone support (1.77 mmol·g^−1^ → 1.54 mmol·g^−1^). In spite of this, the measured amount of leached copper was found to be relatively modest, with reported levels in selected isolated triazole products ranging between 37 ppm and 46 ppm.

### 3.3. Linear Organic Polymer Supports

In the course of their investigation of new Cu(II) complexes with ferrocene-containing Schiff-base ligands, Liu et al. developed a molecularly-enlarged CuAAC pre-catalyst **40** anchored to linear poly(methyl methacrylate) (PMMA) [181].

**Scheme 20:**
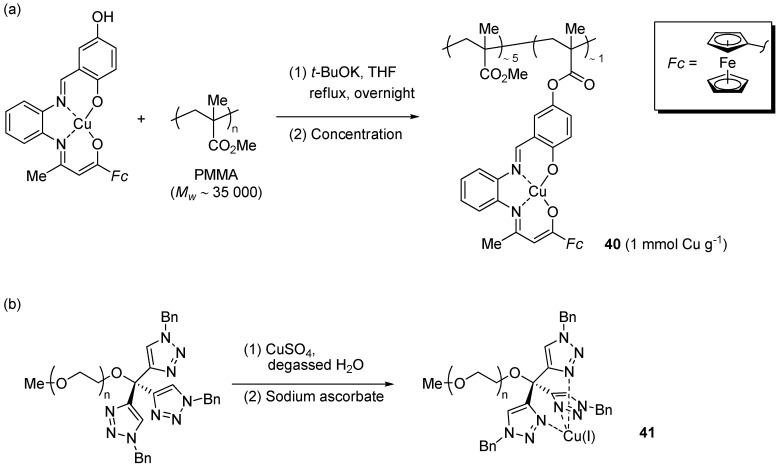
Preparation of the molecularly enlarged Cu(II) pre-catalyst **40** (**a**) and the water-soluble Cu(I) complex **41** (**b**).

The material was obtained by coupling PMMA to the pre-formed complex by trans-esterification (Scheme 20a) and, thanks to its poor solubility in EtOH, was employed as a heterogeneous catalyst in CuAAC runs in this solvent.

Two-component reactions (Scheme 1), for 24 h at r.t. with 2 mol % of **40** reduced with sodium ascorbate, led to nearly constant results in the course of three consecutive reaction cycles (approximate 88% or 95% yields, depending on the specific alkyne substrate). Moreover, efforts directed to address the issue of metal leaching by TEM (detection of metal nanoparticles) and ^1^H-NMR (complexation-induced shift by Cu(I/II) salts) did not provide any clue about the occurrence of such a phenomenon. No attempt was reported, however, for quantifying the copper content by a technique, e.g., ICP-AES, better fit for accurate determinations at the ppm level.

In a very recent example, Wang et al. examined the use of the water soluble complex **41** [78]. Striking features of this system were the possibility to effectively catalyze CuAAC reactions in water down to 10 mol ppm loading level (with TON and TOF values of 86,000 and 3600 h^−1^, respectively) and its suitability for the click modification of dendrimers and substrates of potential pharmaceutical interest (typically at 50–500 mol ppm loading). Because the PEG-containing complex **41** was expected to be poorly soluble in cold diethyl ether, such solvent was employed for recovering the triazole product from the reaction mixture while leaving the catalyst in the aqueous phase. Although the procedure allowed the use of the catalyst solution in 6 consecutive reaction cycles, a significant decrease of product yield was observed (95% → 53%). The problem was attributed to the unavoidable loss of copper ions, due to the strong complexing ability of the heterocyclic nucleus of the triazole product.

### 3.4. Inorganic Supports

**Figure 4:**
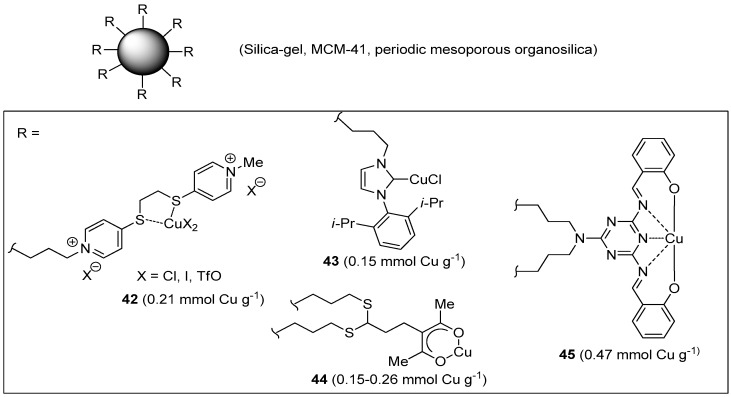
Cu(I/II) complexes with organic ligands covalently immobilized on insoluble inorganic supports.

In addition to the many examples published in the past years [88,89], a few new supported Cu(I/II) complexes have been disclosed recently, which contain low molecular weight organic ligands covalently anchored onto (or within) a siliceous support (Figure 4). Because these materials do not contain any polymeric component, but the oxidic support itself, they will be mentioned only briefly here.

Silica gel decorated with a sulfur-containing ligand was developed by Tavassoli et al. as a new support material for Cu(I/II) [182]. In situ reduction with sodium ascorbate of the complex obtained by stirring the modified silica with CuCl_2_ or Cu(OTf)_2_ afforded the corresponding CuAAC catalyst **42**. Under optimized conditions, which included *inter alia* the use of 0.05–0.1 mol % of the triflate catalyst at r.t. in PEG_400_–H_2_O (1:1), fair to nearly quantitative yields (72%–99%) were obtained in three-component CuAAC reactions (Scheme 2a), after a reaction time comprised between 15 min and 1 h. In the most favorable case, this translated into a remarkable TOF value of 7920 h^−1^. Unfortunately, reuse of the supported system in a series of six benchmark CuAAC runs caused some reduction of product yield (approximate 5%).

Compared to the previous example, the MCM-41 supported Cu(I)-NHC complex **43**, described by Garcés et al. [183], and especially the Cu(I)-acetylacetonate on HMS silica **44**, studied by Lai et al. [184], displayed lower catalytic activity in two- or three-components CuAAC reactions (Scheme 1 and Scheme 2a). In the case of the latter system, however, the material could be used four times in a model reaction, with essentially identical results (99% → 98% yield).

While most of the reported approaches rely on surface functionalization of pre-formed oxide supports, Naeimi et al. resorted to the sol-gel process for immobilizing a copper complex containing trimethoxysilane groups in a templated organosilica matrix [185]. Contrarily to most other studies in the field, testing of the catalytic properties of the material **45** was performed by adopting a Box-Behnken factorial design of experiments in order to optimize an ultrasound-promoted three-component CuAAC reaction in water with styrene oxide (Scheme 2b). The approach led to identify the best conditions in the use of 0.33 mol % of the supported catalyst, under irradiation at 40 kHz for 7 min. By adopting the same experimental set-up, different epoxides could be converted into the corresponding triazole products in fair to excellent yield (74%–99%, TOF up to 2926 h^−1^). In spite of the mechanical stress put on the siliceous material by sonication, the supported system maintained intact its activity in six consecutive runs (98% → 98% yield) and no copper was reportedly detected in the filtrate by AAS.

### 3.5. Magnetically Recoverable Systems

**Scheme 21:**
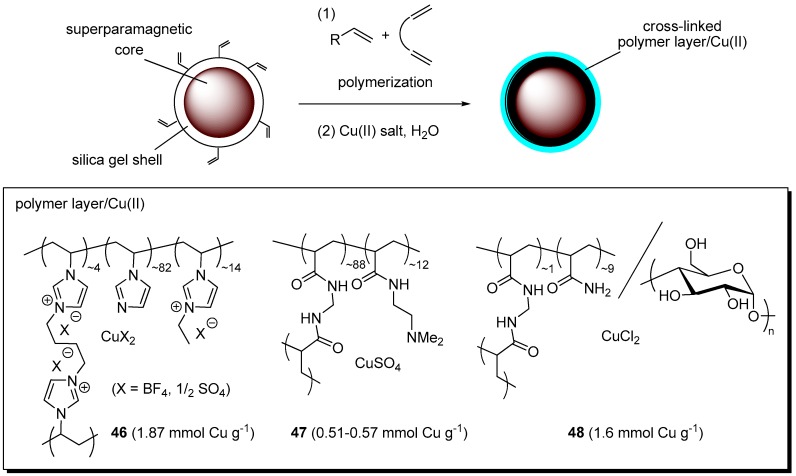
Preparation of supported Cu(II) salts **46**–**48** onto organic cross-linked polymers with super-paramagnetic core.

Similarly to materials containing copper and copper oxide clusters on super-paramagnetic supports (Section 2.6), the last few years saw progresses also for what it concerns magnetically recoverable systems containing Cu(I/II) species on a polymeric organic layer (Scheme 21). Advances in this direction were reported, for instance, by Pourjavadi et al. [186]. With an approach in some respects analogous to that already discussed for the GO-based material **32** (Section 3.1), *N*-vinylimidazole, *N*-ethyl-*N′*-vinylimidazolium tetrafluoroborate, and a dicationic cross-linking agent were subjected to radical copolymerization. In this case the process was carried out in the presence of core-shell Fe_3_O_4_-silica gel nanoparticles bearing surface methacrylate groups, in order to endow the polymeric material (55 wt % by TGA) with a covalently embedded magnetic core. Charging of the resin with CuSO_4_ in the final step led to the supported catalyst precursor **46**. The activity of the material was tested in the three-components CuAAC reaction of benzyl bromide, with sodium ascorbate as the reducing agent. Compromise conditions for minimizing both catalyst loading and reaction time were determined to be the use of 0.75 mol % of supported copper complex in water at 50 °C. With this choice, different chlorides or bromides and terminal alkynes, either aliphatic or aromatic (Scheme 2a), could be effectively coupled in 2–4 h (80%–97% yield). Filtration of the mixture was confirmed to halt completely the reaction progress and ICP-AES assay of the filtrate did not detect any significant concentration of copper. Still, a moderate reduction of turnover frequency was recorded in the course of 6 recycle runs of the benchmark reaction, which forced to increase the time from 150 min to 170 min in order to maintain the product yield in the range 90%–95%. As in other cases, the trend was related to mechanical losses of the supported system during the separation and washing steps.

A similar approach was followed by Zohreh et al. for the preparation of magnetically recoverable nanoparticles consisting of a Fe_3_O_4_ core embed into silica gel and cross-linked poly(methyl acrylate) (PMA) shell layers [187].

Aminolysis of the ester groups of the latter with *N,N*-dimethylethylenediamine allowed the installation of chelating units, exploited in the last step for adsorbing CuSO_4_ from an aqueous solution. When tested in three-components CuAAC reactions (Scheme 2a), the resulting supported complex **47** afforded best results in water at 50 °C, at 0.3 mol % loading and in the presence of an excess of sodium ascorbate. Under these conditions, different triazole products were smoothly formed from the corresponding alkyne and alkyl chloride, bromide or tosylate (75%–97% yield in 1.5–4 h). While no ICP-OES detectable metal was found in the liquid phases from seven consecutive reaction runs, analysis of the magnetic material recovered in the last cycle indicated a slight reduction of the copper content with respect to the original system (0.54 vs. 0.57 mmol·g^−1^). In addition, some mass loss of the nanoparticles as a whole was recorded. As suggested, this fact may represent the likely explanation of the progressively longer reaction time required for attaining constant conversion in consecutive CuAAC runs. Still, it cannot be excluded that metal leaching might play a role in the problem, as seemingly supported by the fact that TOF values corrected for mass loss underwent also some decrease on passing from the first to the last reaction cycle (160 h^−1^ → 142 h^−1^).

Coating of Fe_3_O_4_-silica gel core-shell magnetic nanoparticles with a cross-linked organic polymeric layer was tackled also by Pourjavadi et al. [188]. In this case, the macromolecular material was obtained by copolymerization of acrylamide and *N,N′*-methylenebisacrylamide, in the presence of starch and support nanoparticles bearing methacrylate groups on the surface. Loading with CuCl_2_ afforded the insoluble catalyst precursor **48**, which was examined in three-components CuAAC reactions of halides (Scheme 2a). With sodium ascorbate as the reducing agent, the use of 0.5 mol % of the system in water required to warm the reaction mixture to 50 °C for obtaining the triazole products in good yield after a short reaction time (82%–99% in 1–5 h). A standard ‘filtration experiment′ and ICP-OES determination of copper in the separated reaction mixture excluded significant amounts of metal in the solution. At the same time, the reuse of recovered magnetic nanoparticles revealed a reasonable durability of the catalyst. Indeed, while a step drop of average turnover frequency was observed on passing from the 5th to the 6th reaction cycle (TOF approximate 400 h^−1^ → 300 h^−1^), no further reduction of turnover rate was recorded in the next five runs. Because FT-IR and ICP-OES data for the recovered catalyst were not very dissimilar from those of the original material, the trend was attributed to mechanical losses during the isolation step.

**Figure 5:**
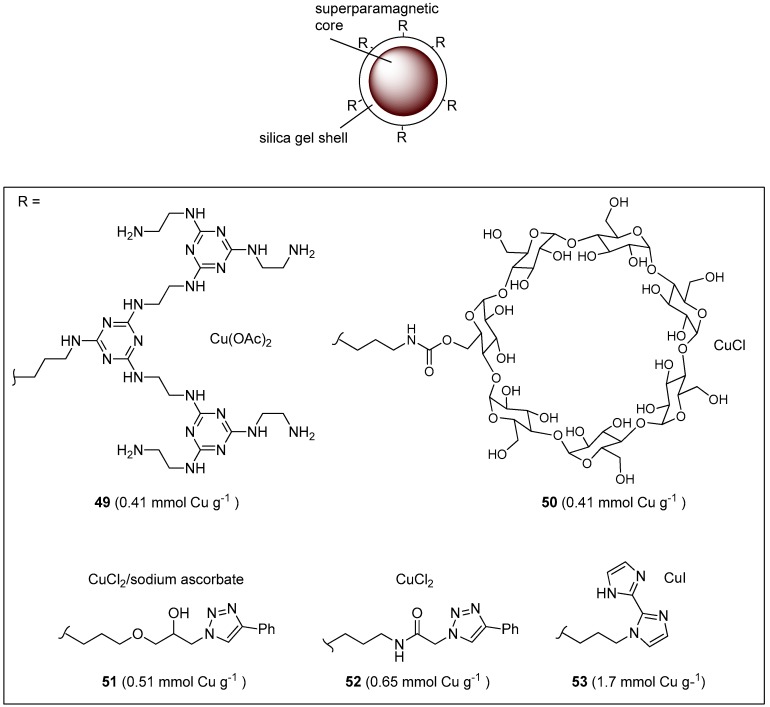
Cu(I/II) complexes **49**–**53**, containing organic ligands immobilized on magnetically recoverable supports.

At the borderline between low molecular weight ligands and high-polymeric ones, functional dendrimers have proved to be very effective in the development of recoverable catalytic systems [189], including those for CuAAC [77]. Very recently, Bahrami and Arabi described the preparation of a triazine-based, 2nd generation dendrimer-Cu(II) complex on ferromagnetic nanoparticles **49** (Figure 5) and its use in three-component CuAAC reactions [190]. The material was obtained by initially coating the Fe_3_O_4_ nanoparticles with an aminopropylsilica layer and then reacting the surface functional groups with trichlorotriazine and ethylenediamine, in a two-round iterative fashion. Although these solid-phase steps were found not to be complete, the final material showed an appreciable adsorbing capacity towards Cu(OAc)_2_ and eventually provided the supported Cu(II) complex as a dark-green solid. Examination of the powder by various techniques (SEM, XRD, and TEM) revealed the presence of well dispersed particles, uniform in shape and size (35 nm average diameter) and consisting of a Fe_3_O_4_ core coated by a thinner silica shell.

The catalytic activity of **49** was optimized in the reaction between benzyl chloride, sodium azide, and phenylacetylene, rather unusually carried out in the presence of an equivalent amount of Na_2_CO_3_. In spite of the relatively low catalyst loading (mostly 0.3 mol % of Cu), nearly quantitative yields were recorded in 10–15 min at r.t. in EtOH-H_2_O (1:1). The same conditions were therefore adopted for the CuAAC of other alkynes and halides (Scheme 2a), to give the corresponding triazoles with excellent yield in no more than 40 min (90%–99%). Surprisingly, this held true not only in the case of very reactive halides (benzyl, allyl, and α-halocarbonyl chlorides and bromides) but even for a reactant, like hexyl bromide, which is expected to undergo the halogen/azide exchange at much slower rate. Recycling of the nanoparticles by magnetic decantation showed some slight reduction of activity along six consecutive runs in the aforementioned benchmark relation (99% → 93% yield). Such a trend was tentatively assigned to “the normal loss the catalyst during the work-up stage”, but no clue was provided about the possible relevance of missing nanoparticles for the problem of product contamination.

**Scheme 22:**
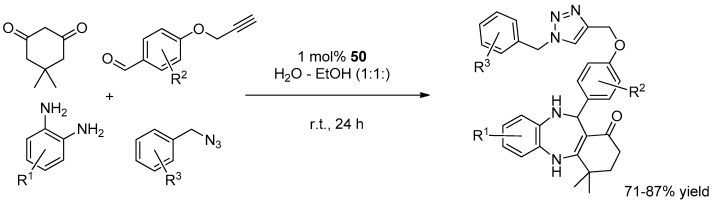
Four-component condensation-click process promoted by **50**.

Also for this class of recoverable CuAAC catalytic systems, examples were reported recently of materials embedding discrete organic ligands (Figure 5). Considering that cyclodextrins (CDs) have found use in CuAAC reactions, with probable roles ranging from phase-transfer agents [191], to ligands for Cu(II) or Cu(I) [192,193], Mahdavi et al. used a surface silanization approach for binding β-CD units to super-paramagnetic iron nanoparticles [194]. Treatment of the resulting white material with CuCl under alkaline conditions afforded a dark powder **50** that, although hard to assess for what it concerns oxidation state, aggregation degree, and location of Cu centers, displayed satisfactory catalytic activity in a four-components reaction involving a CuAAC click step (Scheme 22). Reuse of the magnetically-recovered nanoparticles led to nearly constant yields in the course of 5 cycles and a cumulative TON of 8300 after 10 runs (average TOF = 347 h^−1^). A ‘filtration experiment’, in which the supported catalyst was stirred with EtOH–H_2_O for 24 h revealed a very low metal concentration in the liquid phase (<1 ppm by ICP-AES) that, expectedly, did not lead to any appreciable conversion when standard reactants of the process above were added to the solution. Nonetheless, the fact that in some documented cases copper leaching was significantly larger in the presence of CuAAC substrates and products, than in the reaction solvent alone [75], makes it difficult to evaluate the relevance of the experiment for the problem of metal release under actual four-component reaction conditions.

Given the strong tendency of triazoles to bind copper, the same structural motif has been exploited in the past for retaining Cu(I/II) onto an insoluble support bearing tris-triazole units, e.g., of the TBTA type [195]. More recently, Moghaddam et al. examined the use for the same purpose of mono-triazoles provided with an ancillary coordination site [196,197]. Grafting of ligand units was realized either by reaction of surface-anchored functional groups with a preformed triazole derivative, or by generating the heterocycle nucleus in a three-components CuAAC step. In the latter case the resulting material **51** was obtained already charged with Cu(I). Instead, in the former case the supported Cu(II) complex **52** was prepared by stirring the magnetic nanoparticles with CuCl_2_ in water. At 0.5 mol % loading and in the presence of sodium ascorbate, both systems were active in three-component CuAAC reactions in water with alkyl halides or tosylates (Scheme 2a) and afforded the corresponding triazoles in high yield, after 2–8 h at 55 °C (85%–98%). Recycling of the materials proved feasible, with slight reduction of product yield (approximate 5%) in the course of 10 consecutive runs.

Similarly, 2,2′-bis(imidazole) on core-shell Fe_3_O_4_-SiO_2_ nanomagnetite particles was obtained by Tajbakhsh et al. through reaction of the ligand with the chloropropyl-modified support [198]. Loading with different metal salts led to the corresponding complexes, among which that prepared from CuI (**53**) showed the highest activity in three-component CuAAC reactions (Scheme 2a). With the use of 0.85 mol % of this catalyst at r.t., in water or EtOH–H_2_O (1:1), various chlorides, bromides or tosylates were transformed effectively in the corresponding triazole within 0.5–3.5 h (88%–99% yield). Reuse of the magnetically recovered material demonstrated good preservation of its catalytic activity, with a measurable drop of product yield only in the 5th cycle (99% → 93%) (and the attainment of a still respectable 96% yield in the 6th run, carried out with somewhat extended reaction time: 30 min → 40 min). No textural changes in the recovered solid were noticed by electron microscopy, but copper was detected in minute amounts (0.08% of the initial quantity) in the product phase after the 5th run. On this ground, reloading of the magnetic material with CuI was effected, to give a “regenerated” system with the same activity of the original one.

## 4. Cu-Exchanged Clays and Other Inorganic Materials

Although purely inorganic materials fall outside the main focus of the present review, for the sake of completeness recent advances on the topic are only briefly summarized in this section.

ZMS-5 zeolite exchanged with Cu(II) and Co(II) was used by Safa and Mousazadeh in the three-component CuAAC reaction of benzyl bromides and propargyl alcohol, carried out in water under thermal or US-promoted conditions [199]. The most interesting findings of the study were the observation that the mixed Cu/Co system was more effective than an analogous ZMS-5 zeolite exchanged with Cu(II) alone and the fact that, under sonication, the reaction time could be shortened from several hours to 5 min. The possibility was also mentioned of reusing the catalyst eighth times without significant loss of activity. However, no specific data was provided, neither in this respect nor for what it concerns potential leaching issues.

A comparative evaluation of different catalysts for the CuAAC reaction between dipropargylated alloxan and various benzyl azides was undertaken by Dubey et al. [200]. Apart for the nature of the reactants, the study strictly mirrored in methods and conclusions those of Mohammed et al. for three-components CuAAC reactions involving aromatic boronic acids (Scheme 2d) [201]. Among the systems included in the screening, a Cu(II)-exchanged clay proved superior to other Cu(I/II) catalysts examined in the work. Recovery and recycling of the catalyst was possible, but some decrease of the product yield was observed in the course of five reaction cycles with a fixed reaction time of 3 h (96% → 81%).

In their continuing interest in flow-chemistry, Ötvös et al. examined a Cu(II)-Fe(III) layered double hydroxide (LDH) as an heterogeneous catalyst for *in continuo* two-components CuAAC reactions [202]. When the material was used in a stainless-steel reactor provided with a back-pressure regulator set to 100 bar, optimal results (77%–98% yield) were obtained in CH_2_Cl_2_ at 100 °C with contact time as short as 100 s. With this experimental set-up, the system could be kept working for 10 h without any evident decrease in activity and provided the expected triazole product with a final TON around 5 and a residual copper content of 45.3 ppm. Although the resting state of the catalyst was found to be Cu(II), its activity in CuAAC was explained by reasoning that under the vigorous reaction conditions cupric ions might undergo in situ reduction (e.g., by dehydrogenative homocoupling of the alkyne substrate), to generate Cu(I) active sites as defects in the LDH lattice.

González-Olvera et al. carried out a comparison of the catalytic properties in CuAAC of a Cu-Al LDH, with those of the Cu(Al)O mixed oxide obtained by high temperature calcination of the former [203]. The mixed oxide was found to be more active and better fit for recycling than the LDH, especially when calcination was used before each new run in order to get rid of any organic material deposited on its surface. Both materials displayed some tendency to leach copper in the solution, albeit to an extent significantly larger for LDH than for the mixed oxide (0.4% and 4%, respectively). The phenomenon, attributed to the capability of sodium ascorbate to chelate metal ions, translated into an appreciable catalytic activity in the filtrate. This let the Authors to conclude that, at variance with most of the supported CuAAC catalysts reported in related studies, homogeneous and heterogeneous type of catalysis coexisted in their system.

Another instance of a copper-exchanged clay material, in this case natural montmorillonite, was described recently by Mekhzoum et al. [204]. Rather surprisingly in view of the very low solubility of CuI in water (approximate 4.2 × 10^−4^ M at 18–20 °C) [205], the material was reportedly prepared by vigorous stirring of the clay in a saturated aqueous solution of the copper salt. The activity of the system was probed in the two-components CuAAC reactions (Scheme 1), performed in water at r.t. and with about 2 mol % of supported catalyst (88%–98% yield in 2 h). Reuse of the material recovered by filtration proved also feasible, albeit with a slight decrease of product yield in five consecutive cycles (97% → 92%).

Finally, the entrapment of Cu(II) in a silica matrix by sol-gel process allowed Diz et al. to prepare spherical nanoparticles (<100 nm in size) containing variable amounts of the metal (0.05 wt %–9.4 wt %) [206]. Stirring with an aqueous solution of sodium ascorbate led to complete fading of the pale-blue color of the solid and afforded an EPR silent material with reasonable activity in two- and three-components CuAAC reactions in DMF or *t*-BuOH-H_2_O (3:1) (Scheme 1 and Scheme 2a: 80%–98% yield in 3–6 h at r.t., at 5 mol % loading and with three equiv. of added *N,N*-diisopropylethylamine). Recyclability of the catalytic system was verified in two series of 10 consecutive runs (93% → 87% or 89% → 87% yield), while the nature of catalysis was confirmed to be heterogeneous by running not only a standard “filtration experiment”, but also a “three-phase test” with a benzylic azide derivative anchored to Wang resin. Remarkably, when the filtrates from two CuAAC runs were subjected to copper quantification by ICP-MS, a very low metal content was found (approximate 0.1–0.2 ppb). Even if the figures for dry triazole products are at least one order of magnitude larger, it seems therefore that copper contamination by the system under exam is one of the lowest reported to date for any recoverable CuAAC catalyst.

## 5. Conclusions

With a cost ranking among the lowest for transition metals employed in organic synthesis, it might be argued that recovery and recycling of the copper catalyst used in CuAAC is not of prime importance for the economics of the process. Still, the difficulty of removing the metal from highly functionalized products or polymeric materials and the specific problems raised by biomolecules, prompted the introduction of new and more effective protocols in the past years. Almost invariably, this resulted in the use of relatively expensive soluble ligands that in addition could not solve completely the issue of product contamination. Especially in the perspective of synthesizing triazole compounds supposed to undergo biological screening, or whose functional properties (e.g., fluorescence) are adversely affected by copper contamination, this state of facts justifies the efforts currently devoted to the quest for improved CuAAC catalytic systems.

As shown also by the cases discussed in the present review, the proposed solutions range from very simple materials to very complex ones. In the case of the former, once the separation issue is granted and a large enough turnover is achieved, the possibility of reusing the catalyst is probably a useful, but not strictly essential feature of the system. On the contrary, in the case of catalyst comprising expensive components the attainment of extensive recycling (presumably much beyond what disclosed in the examples reported to date) appears to be an additional mandatory requirement, at least in view of their possible use at scale [207].

In this context it is worth of note that, although the need or reducing copper contamination is generally acknowledged in the introductory section of most of the published works, little care is paid in a surprisingly large number of papers to provide reliable data on this aspect. In perspective, with the introduction of additional components in the catalytic system that might be even more toxic (e.g., Ni) or more difficult to spot than copper (e.g., organic contaminants from partially soluble polymeric components in the material), this situation is at risk of taking the research in the field, very far from its stated target.

As noted in the review of Haldon et al., “almost any copper source that delivers catalytically active Cu(I) species in the reaction mixture can be employed as a pre-catalyst in the CuAAC reaction” [88]. Although the same conclusions can be drawn also for the recoverable systems discussed in the present paper, different materials display nonetheless a broad spectrum of activity: Some work at low loading and temperatures close to 25 °C, in the very spirit of click-chemistry, other require high-boiling solvents at reflux temperature. While this allows certainly some kind of ranking between the systems that keep being published, also from this point of view a punctual comparison is severely hampered in some cases by the lack of relevant data like, e.g., catalyst loading.

Considering the degree of ingenuity demonstrated in many of the examples reported to date, the unquestionable success with some of the disclosed systems, and the relevance for the development of greener synthetic processes, further developments in the field of recoverable catalyst for CuAAC are expected. Hopefully, these advancements will strive to take the issues above into greater account and make an effort to adhere more strictly to recommended best practices for benchmarking new catalysts [208].

## Abbreviations

The following abbreviations are used in this manuscript:

AAS	Atomic absorption spectroscopy
AES	Atomic emission spectroscopy
Bipy	4,4′-Bipyridine
CD	Cyclodextrin
CNT	carbon nano-tubes
CP-MASS	Cross-polarization magic angle spinning spectroscopy
CRGO	Chemically-reduced graphene oxide
CS	Chitosan
CuAAC	Copper-catalyzed azide-alkyne cycloaddition
CuNPs	Copper nanoparticles
DMF	*N,N*-Dimethylformamide
DMSO	Dimethylsulfoxide
DVB	divinylbenzene
EDACell	Ethylenediamine-modified cellulose
EDS	Energy-dispersive (X-ray) spectroscopy
FT-IR	Fourier-transform infra-red spectroscopy
GO	Graphene oxide
Himdc	4,5-Imidazoledicarboxylate
HPA	Heteropolyacid
ICP	Inductively-coupled plasma
LDH	Layered double hydroxide
M-dots	Melanine nanoparticles
MOF	Metal-organic framework
MS	Mass spectrometry
MW	Microwave, molecular weight
NHC	*N*-Heterocyclic carbene
OES	Optical emission spectroscopy
pABA	Poly(2-aminobenzoic acid)
PANI	Polyaniline
PIB	Poly(isobutylene)
PEG	Poly(ethylene glycol), poly(ethylene oxide)
Pim	Poly(vinyl imidazole)
p[C4R-NH_2_]	amine-modified calix[4]resorcinarene-formaldehyde resin
P[im/IL]	Imidazole-ionic liquid copolymer
PMMA	Poly(methyl methacrylate)
POM	Polyoxometalate
ppm, ppb	parts per milion, parts per bilion
PTFE	Poly(tetrafluoroethylene)
PVA	Poly(vinyl alcohol)
PVP	Poly(*N*-vinyl pyrrolidone)
PVPh	Poly(4-vinylphenol)
r.t.	Room temperature
SEM	Scanning electron microscopy
SMI	Poly(styrene-*co*-maleic anhydride) imide
SNW	Schiff-base network polymer
TBTA	Tris[(1-benzyl-1,2,3-triazole-4-yl)methyl]amine
TEM	Transmission electron microscopy
TGA	Thermogravimetric analysis
THF	Tetrahydrofurane
TOF	Turnover frequency
TON	Turnover number
UV	Ultraviolet spectroscopy
XPS	X-ray photoelectron spectroscopy
XRD	X-ray (powder) diffraction analysis
XRF	X-ray fluorescence analysis
